# Oxygen Supply of Islets of Langerhans by Photosynthetically Active Microalgae in Bioprinted Co‐Cultures Maintains Their Function in a Hypoxic Environment

**DOI:** 10.1002/adhm.202505927

**Published:** 2026-03-08

**Authors:** Finn Dani, Sarah Duin, Ashwini Rahul Akkineni, Susann Lehmann, Barbara Ludwig, Michael Kühl, Michael Gelinsky, Anja Lode

**Affiliations:** ^1^ Centre for Translational Bone, Joint and Soft Tissue Research, University Hospital Carl Gustav Carus and Faculty of Medicine Technische Universität Dresden Dresden Germany; ^2^ Paul Langerhans Institute Dresden of Helmholtz Centre Munich and Department of Medicine III, University Hospital Carl Gustav Carus Technische Universität Dresden Dresden Germany; ^3^ Marine Biological Section, Department of Biology University of Copenhagen Helsingør Denmark

**Keywords:** bioprinting, co‐culture, diabetes, insulin, microalgae, oxygen, pancreatic islet transplantation

## Abstract

Type 1 diabetes mellitus (T1D) is characterized by the autoimmune destruction of pancreatic beta cells, leading to insulin deficiency and necessitating lifelong external insulin administration. The transplantation of allogenic islets is a promising therapeutic approach, whereby their macro‐encapsulation offers immune protection but restricts oxygenation after transplantation. This study addresses the challenge of oxygen supply by developing a spatially structured co‐culture system using bioprinting, in which both pancreatic islets and the photosynthetically active microalga *Scenedesmus* sp. are embedded in alginate‐based hydrogels. Key environmental parameters for long‐term co‐cultivation were developed and systematically optimized: red light illumination was identified as non‐detrimental to islet viability and function while supporting microalgal photosynthesis at the same time, and a co‐culture medium was formulated to fulfill the metabolic requirements of both cell types. In direct co‐culture experiments under hypoxic conditions, microalgae generated sufficient oxygen to maintain normoxic conditions, thereby preserving islet viability and glucose‐stimulated insulin secretion over several days. The results demonstrate that spatially organized bioprinting enables the close proximity of islets and microalgae, facilitating effective oxygen transfer in vitro. This work establishes a robust framework for functional mammalian–microalgae co‐cultures, optimizing conditions to reliably maintain cell health and function through photosynthetically generated oxygen.

## Graphical abstract

This study developed a bioprinted co‐culture system embedding rat pancreatic islets and Scenedesmus sp. microalgae spatially defined in close vicinity. Red light was found optimal to ensure microalgal photosynthesis while maintaining islet viability and functionality. A tailored co‐culture medium supported both cell types. In hypoxic conditions, microalgae generated sufficient oxygen to maintain islet viability and insulin secretion, demonstrating effective oxygen transfer via spatially organized bioprinting.

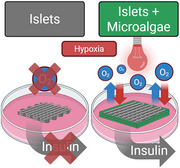

## 1 Introduction

Type 1 diabetes mellitus (T1D) is an autoimmune disease that affects more than eight million people worldwide. Patients are required to closely monitor their blood glucose levels, but even with modern medicine, the life expectancy of patients with T1D is reduced. The current treatment of T1D primarily revolves around the administration of external insulin, as the mechanism of this disease, the autoimmune destruction of pancreatic beta cells, leads to an absolute insulin deficiency, which is deadly when not treated [1]. The administration of insulin can be achieved through various methods, including multiple daily injections or continuous subcutaneous insulin infusion via insulin pumps. Mimicking physiological insulin secretion is crucial for achieving optimal glycemic control and minimizing complications associated with diabetes, but remains difficult even with state‐of‐the‐art technologies, so most patients still suffer from long‐term complications later in life [2, 3].

The transplantation of islets of Langerhans containing the insulin‐producing beta cells is a promising therapeutic approach for managing T1D, particularly for patients who frequently experience severe glycemic fluctuations or have difficulty achieving glycemic control with insulin therapy alone. Islets of Langerhans of an allogenic organ donor are transplanted into the patient, typically via the portal vein into the liver. Upon transplantation, the islets temporarily lose their vascularization, leading to a decreased insulin release from the transplanted islets as they are highly sensitive to hypoxic conditions [4, 5]. Studies indicate that up to 80% of transplanted islets may undergo cell death shortly after transplantation due to hypoxia and inflammatory responses [6, 7, 8].

A further major limitation of allogenic islet transplantation is the necessity for immunosuppression to prevent immune rejection and to enhance the lifespan and function of the transplanted cells. Encapsulating the islets in hydrogels isolates them from the recipient's blood stream, and thereby the immune system, preventing severe side effects associated with immunosuppressants, while extending the lifespan of the implants [6, 9]. Furthermore, immunoisolation facilitates the use of xenografts (e.g., porcine islets), alleviating or overcoming the issue of limited human donor availability [10, 11]. However, a key challenge with this approach is that isolation from the blood stream compromises the nutrient supply of the embedded islets, which is particularly problematic in the case of oxygen [12, 13].

Encapsulation in alginate‐based hydrogels is a prominent area of research due to the biopolymer's non‐immunogenic properties when sufficiently purified, as well as its widespread application attributed to its ease of handling and versatility in various biomedical contexts [14, 15]. Encapsulating a single or a few islets (micro‐encapsulation) ensures short diffusion distances, but the small droplets increase the total volume that needs to be transplanted and make the removal of the transplanted islets more difficult in case of a loss of function. Therefore, encapsulating a large number of islets in a macroscopic alginate structure (macro‐encapsulation) can greatly improve handling. A successful macro‐encapsulation of pancreatic islets in clinical‐grade alginate was demonstrated in the “beta‐Air” system, consisting of two compartments, one for immobilized islets and, separated from this by a gas‐permeable membrane, an oxygen reservoir. The system was encased in a plastic housing to increase stability and included a membrane to facilitate mass transport between the implant and the recipient's body. In a human trial, this implant has been shown to provide, in addition to immunoprotection a sufficient oxygen supply, retaining functional islets for 10 months [16, 17, 18]. However, the reservoir had to be refilled daily via subcutaneous ports to ensure sufficient oxygenation of the islets. This leads to significant restrictions on patient activity; in addition, the oxygen reservoir connection is associated with a risk of infection.

An exciting alternative concept for oxygen supply independent of external sources is the exploitation of photosynthesis for local oxygen generation. Initial studies, conducted with the photosynthetically active microalga *Chlorella sorokiniana*, demonstrated that the microalgae produced sufficient oxygen to ensure survival and maintain the function of beta cells immobilized in alginate for a short duration of up to 3 h under anoxic conditions [19, 20]. The first longer‐term application was demonstrated by co‐implanting the photosynthetically active cyanobacteria *Synechococcus lividus* with islets into the back of diabetic rats. The two cell types were separated by a gas‐permeable membrane, and the entire chamber was surrounded by an immunoisolating membrane. The islets remained viable and functional for one month and stabilized the blood‐glucose levels in the rats. However, the number of implanted islets was not clinically relevant because only a low islet density could be supported by the generated oxygen. To achieve islet numbers relevant for human patients, photosynthetic efficiency and diffusion between the oxygen‐producing and oxygen‐consuming compartments need to be improved [21].

In recent years, co‐culture systems involving mammalian cells and photosynthetically active microalgae have garnered significant attention to solve the problem of insufficient oxygen supply. However, the development and implementation of such systems face several challenges, primarily arising from the contrasting physiological and environmental requirements of these phylogenetically distinct organisms. One critical issue is scalability; ensuring consistent and efficient exchange between mammalian cells and microalgae becomes increasingly difficult as culture volumes increase. Furthermore, the supply and distribution of light — fundamental requirements for photosynthesis — pose significant technical hurdles. Inadequate light penetration in larger systems can create hypoxic zones, which compromise the survival and performance of both cell types. Additionally, competition for nutrients within the shared medium presents a persistent challenge. Microalgae may outcompete mammalian cells for essential nutrients, while substances produced by one organism, such as metabolic byproducts, can inhibit the growth or function of the other. Therefore, careful design and optimization of such systems are necessary to achieve the delicate balance required for successful co‐culture operations [22, 23, 24].

To enhance the control over the interactions between the extremely different cell types in such co‐cultures, and to improve the oxygen exchange, our group has proposed the application of 3D bioprinting [25]. This technique involves the layer‐by‐layer deposition of cell‐laden hydrogels, known as “bioinks,” to create volumetric constructs with defined geometry and macroporous structures. By embedding cells within the hydrogel during fabrication, this method allows for efficient and spatially precise patterning of distinct cell functions. Thus, this approach allows for the separate incorporation of mammalian cells and microalgae in adjacent hydrogel strands, optimizing the oxygen transfer while minimizing direct interaction between the cell types and preventing local hypoxic zones. By using extrusion‐based bioprinting, both microalgae [25, 26] as well as rat and porcine islets [27, 28] were successfully embedded in alginate‐based hydrogels, retaining their viability, morphology, and functionality.

The objective of the present study is to develop a bioprinted co‐culture system consisting of islets of Langerhans and photosynthetically active microalgae, cultivated together in one construct. Bioprinting macroporous constructs ensures short diffusion distances and promotes an efficient gas exchange between the microalgae and the islets, which are separately encapsulated in adjacent hydrogel strands. By investigating the viability and functionality of rat islets in these co‐cultures under hypoxia, we aim to provide the proof‐of‐principle that photosynthetically active microalgae can supply the islets with oxygen in such a co‐culture system.

## 2 Results and discussion

### 2.1 Concept and Components of the Co‐Culture System

Since islets of Langerhans and microalgae have vastly different environmental and nutritional demands, several parameters needed to be addressed in order for both cell types to survive and retain their function. Mammalian cells have a narrow window of preferred culture conditions, based on the conditions in the (human) body: They require a temperature of 37°C as well as a cell culture medium with glucose as the main organic carbon source and other organic components as essential nutrients. Mammalian cells are typically not exposed to light. On the other hand, microalgae are able to adapt to diverse environmental conditions but need light to perform photosynthesis and thereby act as natural oxygen generators. Therefore, key factors for establishing a successful functional co‐culture were defined as (1) selecting a suitable microalgae partner that is adapted to 37°C, (2) implementing an illumination regime that facilitates algal photosynthesis without adversely affecting mammalian cells, and (3) developing a growth medium that supports the functionality of both cell types over several days.

**FIGURE 1:**
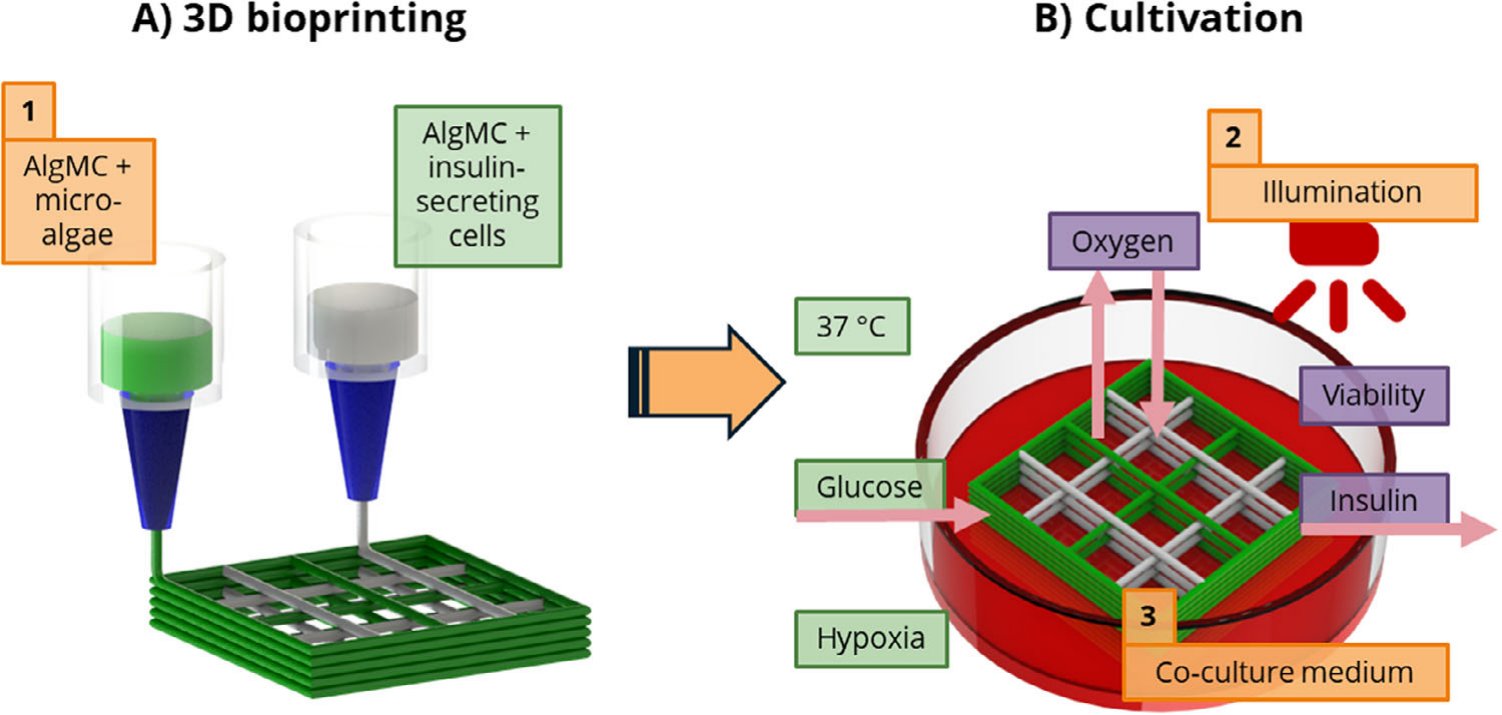
Schematic of the concept, components, and influencing factors of the bioprinted co‐culture. (A) Multi‐channel extrusion bioprinting is applied to fabricate co‐culture constructs by alternating deposition of microalgae‐laden and islet/beta cell‐laden hydrogel strands. Both bioinks are based on an alginate‐methylcellulose (AlgMC) blend. (B) Under illumination, immobilized microalgae produce oxygen that is consumed by the neighboring islets/beta‐cells, which secrete insulin in response to glucose. The fixed parameters of the co‐culture system are shown in green, and depicted in orange are the parameters that need to be investigated and established in order to achieve the desired results, whereby these results — viable cells, stable oxygen supply, and insulin secretion — are shown in purple.

Figure 1 depicts the concept of the proposed co‐culture system. In the first step, shown in subfigure A, the insulin‐producing beta‐cells and a suitable microalgae partner are immobilized into one construct via extrusion‐based 3D bioprinting of the two bioinks, both based on a blend of 3 wt.% alginate and 9 wt.% methylcellulose (AlgMC) [25, 27]. After crosslinking, the bioprinted constructs were subjected to cultivation (subfigure B). Here, a temperature of 37°C and the presence of glucose were dictated by the mammalian cell partner, while hypoxic conditions were chosen to mimic the situation after islet transplantation in a macrocapsule.

The key factor 1 – a suitable microalgae partner – was selected in a previous study in which we investigated four thermotolerant microalgae strains regarding parameters that are essential for mammalian cell culture: In bioprinted constructs, the green microalga *Scenedesmus* sp. displayed a high photosynthetic activity at a temperature of 37°C under white and red‐light illumination. Further, *Scenedesmus* sp. retained a mainly autotrophic metabolism in the presence of glucose [29]. The key factors 2 (illumination) and 3 (coculture medium) had to be adapted for the cultivation phase; viability of both cell types, oxygen content of the medium, and insulin secretion were determined to assess the success of the co‐culture. They were investigated in the present study and are discussed in the following chapters. The murine beta cell line INS‐1 [30] was selected for preliminary tests to establish the key factors 2 and 3, before all findings were verified by using primary rat islets. Finally, the interactions between both cell types were investigated in a proof‐of‐concept co‐culture experiment.

### 2.2 Influence of Light

Light is essential for plants and microalgae to perform photosynthesis. However, the natural, sun‐like white light illumination that most microalgae are adapted to is detrimental to most mammalian cells that are naturally not exposed to light, so a different kind of illumination is necessary for a successful co‐culture.

Red light, which has the longest wavelength within the visible light spectrum, carries less energy compared to white light. As a result, it does not adversely affect mammalian cells [31, 32, 33]. In an initial study, we evaluated the effects of long‐term, continuous exposure to various wavelengths of light — specifically white, blue, and red — on 2D cultures of different mammalian cell types, including the beta cell line INS‐1. Our findings indicated that red light had no detrimental effects on the viability, growth, and morphology of the cell types examined. Additionally, it also promoted the growth and photosynthetic activity of the chosen microalgae partner, *Scenedesmus* sp., both in suspension culture and in bioprinted constructs [29, 34].

In the present work, we investigated the influence of red (590–660 nm wavelength) and white (410–780 nm wavelength) light on bioprinted samples of the model cell type INS‐1 and validated our findings regarding viability and functionality with bioprinted islets of Langerhans.

**FIGURE 2:**
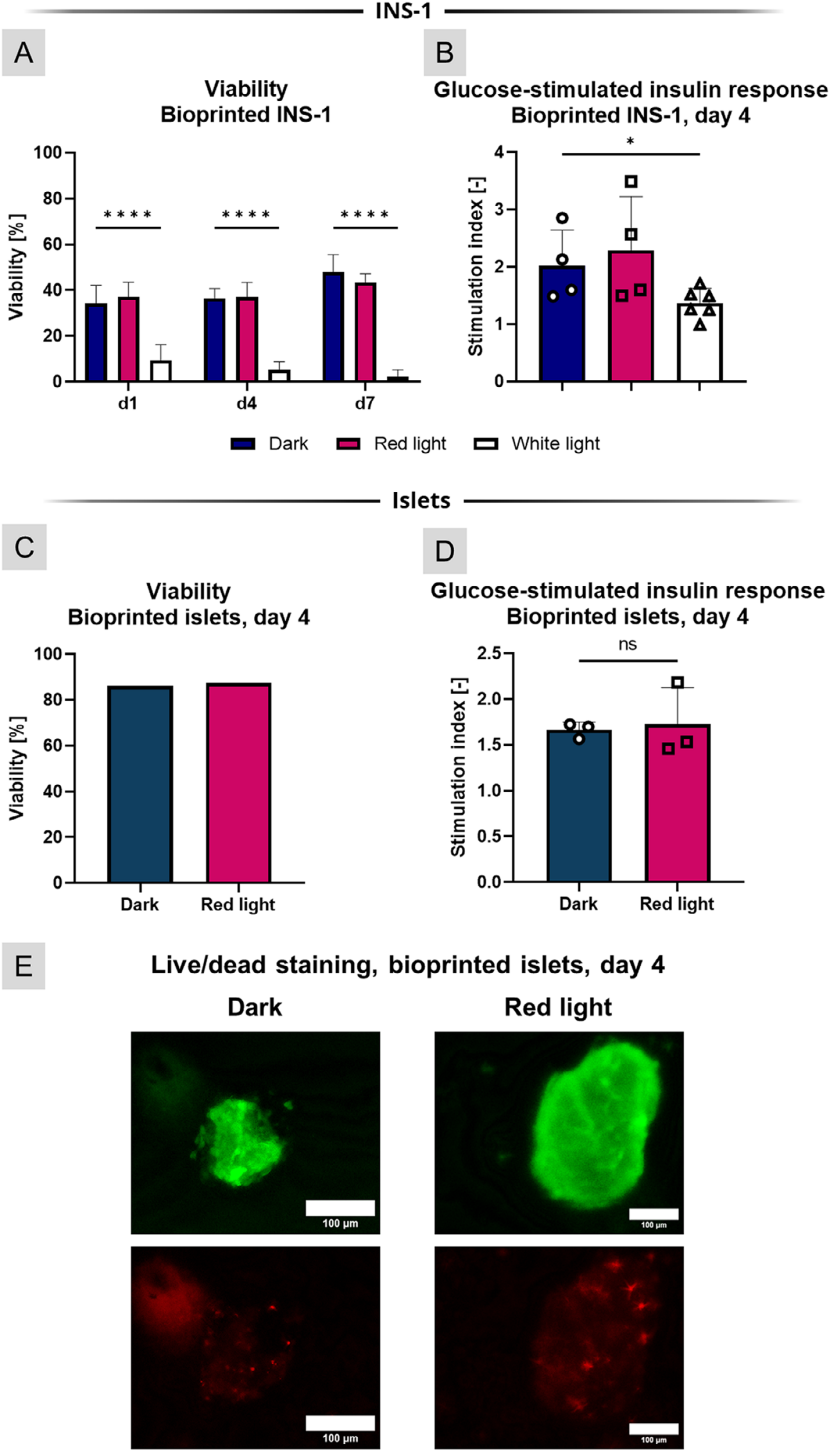
Influence of light on bioprinted INS‐1 cells and rat pancreatic islets. (A) Viability (n≥12) over 7 days of cultivation and (B) glucose‐stimulated insulin response on day 4 of cultivation (n≥4) of INS‐1 cells, which were either cultivated in the dark (control), or under red‐light or white‐light illumination in their standard RPMI 1640‐based culture medium. (C) Viability (n = 8) and (D) glucose‐stimulated insulin response (n = 3) of rat islets after 4 days of cultivation in the dark or under red‐light illumination in their standard RPMI 1640‐based culture medium. Stimulation index is calculated by dividing the amount of insulin secreted in high glucose conditions (16.4 mmol L^−1^) by the amount of insulin secreted in low glucose conditions (3.3 mmol L^−1^) and serves as reference to compare functionality of insulin‐secreting cells. (E) Representative images of viability staining of rat islets (green: live cells, red: dead cells; scale bars: 100 µm). Statistical significance: ^*^ *p* < 0.05, ^****^ *p* < 0.0001.

INS‐1 were printed in AlgMC, and after crosslinking, the constructs were cultivated in the standard RPMI‐based medium with continuous red or white‐light illumination for a period of seven days, during which their viability and glucose‐stimulated insulin response (GSIR) were analyzed (Figure 2A,B). GSIR describes the stimulation of insulin‐producing cells to assess the reactivity of the cells to varying glucose levels, as described in chapter 4.7. Briefly, insulin‐producing cells are immersed in a buffer solution with a low glucose content. After a set time, the solution is removed and replaced with a buffer solution with a high glucose content. Then, the insulin content of both supernatants is analyzed to observe the changes in the cells’ insulin secretion in reaction to the available glucose. To measure the functionality of the cells, the amount of secreted insulin in high glucose conditions is then divided by the amount of secreted insulin in low glucose conditions, resulting in the Stimulation Index (SI).

While INS‐1 illuminated by red light did not show any reduction in viability compared to the control group cultivated in the dark, INS‐1 illuminated by white light presented a significantly lower viability rate, with only 9 % living cells on day one and almost no living cells detectable on day seven. This pronounced effect was not found when analyzing the GSIR on day four: While there was a slight trend toward lower numbers on the Stimulation Index (SI), no statistically significant differences were observed (2.0 in the dark, 2.3 under red‐light illumination, and 1.4 under white‐light illumination). Thus, these results indicate that continuous red‐light illumination does not have a detrimental effect on the viability or functionality of bioprinted INS‐1 cells. However, white light negatively impacted the viability of bioprinted INS‐1 significantly, which corroborates our previous results regarding the influence of light on 2D cultures of INS‐1 [34]. Despite this, similar SI values suggest that the surviving cells, even under white‐light illumination, retained their functionality.

For the islet experiment, pancreatic islets from twelve 22‐week‐old female Wistar rats were isolated, and ∼4500 islet equivalents (IEQ) were bioprinted according to the protocol described in the experimental section; the results are shown in Figure 2C–E. The viability of the islets was determined by manually categorizing the fluorescence images as described in Karaoz et al. [35]: therefore, no standard deviation was calculated. Under red light, the viability of the islets was comparable to the viability in the dark control group, with around 86% to 87.5% living cells. Additionally, there were no visible signs of necrotic cores or morphological changes detected using fluorescence microscopy (see also Figure S3). The stimulation index for islets under red light was also similar to the dark control group (1.66 and 1.72, resp.). Other studies involving co‐cultures with islets under illumination did not provide data on the influence of light on islets; either the cultivation time was too short [20] or no light reached the islets [21]. Yet, in the context of photobiomodulation – a safe and clinically applied approach to stimulate mammalian cells across various medical fields, including diabetes treatment – red light is highly effective due to its deep tissue penetration and shows no negative effects on pancreatic rat islets when illuminated for up to 24 h [36, 37].

### 2.3 Development of a Co‐Culture Medium

#### 2.3.1 Composition of the Co‐Culture Medium

The development of a co‐culture medium (CCM) was mandatory for the in vitro investigation of the proposed co‐culture. Given that both cell types have vastly different requirements regarding their nutrients, a medium composition needed to be developed that allowed for the autotrophic growth of microalgae and the heterotrophic growth of mammalian cells. Consequently, the developed medium should closely mimic the composition of their respective standard media and contain all essential components.

The standard media of INS‐1 and rat islets consist of the basal RPMI 1640 medium and essential supplements such as heat‐inactivated fetal bovine serum (detailed compositions shown in Tables S1 and S2). The used microalgae medium (TP medium) is a TRIS‐phosphate buffer solution supplemented with a salt solution (MgSO_4_, CaCl_2_, NaNO_3_) and Hutner's trace elements (Tables S3 and S4).

As the media supplements are essential, their original concentrations for both cell types (as detailed in the materials section and Table 1) were maintained in the CCM to support growth and function as much as possible. In preliminary tests, we examined potential adverse effects of the supplements from either medium on the other cell type. The INS‐1/islet media supplements (FBS, sodium pyruvate, glutamine, 2‐mercaptoethanol) did not affect the viability and growth of the microalgae. Vice versa, the trace element solution as microalgae supplement did not negatively influence the viability and proliferation of mammalian cells, but the NH_4_Cl_2_ present in the original salt solution had to be substituted by NaNO_3_ to avoid a toxic effect. Therefore, we also altered the TP medium used as reference medium in this work, as described previously [29]. The basis of the CCM is a mixture of the RPMI 1640 medium and TP medium. Preliminary tests revealed that a ratio of 1:1 resulted in a significant reduction in viability and proliferation of mammalian cells, while the replacement of ¼ RPMI with TP medium was acceptable for both cell types. Thus, a 3:1 mixture was used in the CCM that did not excessively dilute the medium for the mammalian cells. Table 1 gives an overview about the composition of the CCM for INS‐1/*Scenedesmus* sp. and islet/*Scenedesmus* sp. co‐cultures, which were used for the following experiments. In a previous study, we already assessed the influence of antibiotic supplements on *Scenedesmus* sp. and identified the semisynthetic antibiotic ampicillin as an optimal replacement for the microalgae‐toxic penicillin/streptomycin combination [29].

**TABLE 1:** Components of the co‐culture media (CCM) for INS‐1/Scenedesmus sp. and rat islets/Scenedesmus sp. combinations, given in vol%. Black: components originating from INS‐1/islets standard media [27, 30]; green: components originating from the modified TP medium for microalgae [29]; TP medium components: see Table S4.

CCM for INS‐1 and *Scenedesmus* sp.		CCM for rat islets and *Scenedesmus* sp.	
64.35%	RPMI 1640 (11.1 × 10^−3^ м glucose)	65.44%	RPMI 1640 (no glucose)
10%	HI‐FBS	10%	HI‐FBS
1%	0.1 m Sodium pyruvate/ 5 × 10^−3^ м 2‐mercaptoethanol		—
1%	0.2 м L‐glutamine		—
2%	1 м HEPES	2%	1 м HEPES
	—	0.55%	1 м Glucose
20.70%	dH_2_O	21.04%	dH_2_O
0.43%	1 m TRIS‐base	0.44%	1 m TRIS‐base
0.21%	Salt solution	0.22%	Salt solution
0.0022%	Phosphate buffer	0.0218%	Phosphate buffer
0.1%	Hutner's trace elements	0.1%	Hutner's trace elements
0.2%	50 mg mL^−1^ Ampicillin	0.2%	50 mg mL^−1^ Ampicillin

### 2.4 Effect of the co‐culture Medium on Insulin‐secreting Cells

**FIGURE 3:**
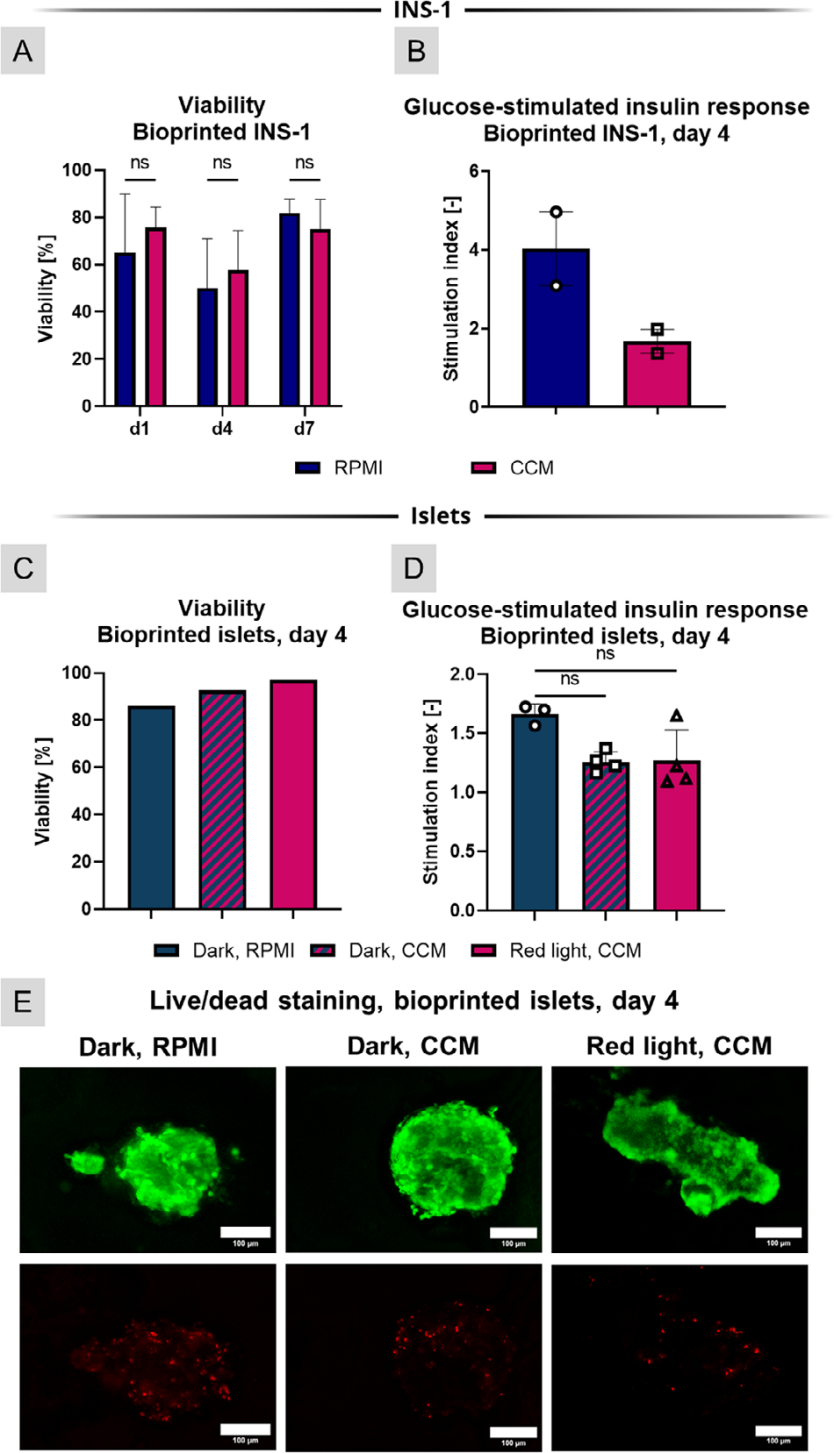
Influence of the co‐culture medium (CCM) on bioprinted INS‐1 cells and rat pancreatic islets. (A) Viability (n≥6) over 7 days of cultivation and (B) glucose‐stimulated insulin response on day 4 of cultivation (n = 2) of INS‐1 cells cultured in the dark in either their standard RPMI 1640‐based culture medium or in CCM (ns: not significant). (C) Viability (n≥6) and (D) glucose‐stimulated insulin response (n≥3) of rat islets after 4 days of cultivation in the dark in either their standard RPMI 1640‐based culture medium or in CCM, as well as under red light illumination in CCM (ns: not significant). (E) Representative images of viability staining of islets (green: live cells, red: dead cells; scale bars: 100 µm). n.s.: statistically not significant.

The effect of the CCM was first investigated on bioprinted INS‐1 (Figure 3A,B). Both the control group in standard RPMI medium and the group cultivated in CCM presented rather high standard deviations, but there were no significant differences in viability rates over seven days of cultivation. The glucose‐stimulated insulin response was decreased in CCM (4.0 in RPMI, 1.7 in CCM) but remained above 1, that is, above the threshold indicating functionality.

For the islet experiment, again, islets from twelve 22‐week‐old female Wistar rats were isolated and bioprinted. Figure 3C,D represent the influence of the islet CCM on islets, cultivated for four days in the dark or under red‐light illumination. In all cases, viability remained well over 80%, with islets cultivated in CCM under illumination showing the highest survival rate with 97%. The GSIR, similar to that of the INS‐1, revealed a slightly lowered SI when islets were cultivated in CCM, but again they remained functional (1.7 in RPMI, 1.3 in CCM, both in dark and red‐light conditions). Neither the CCM nor the combination with red light led to any changes in morphology or a higher prevalence of necrotic cores (Figure 3E; Figure S3).

### 2.5 Effect of the Co‐Culture Medium on Microalgae

**FIGURE 4:**
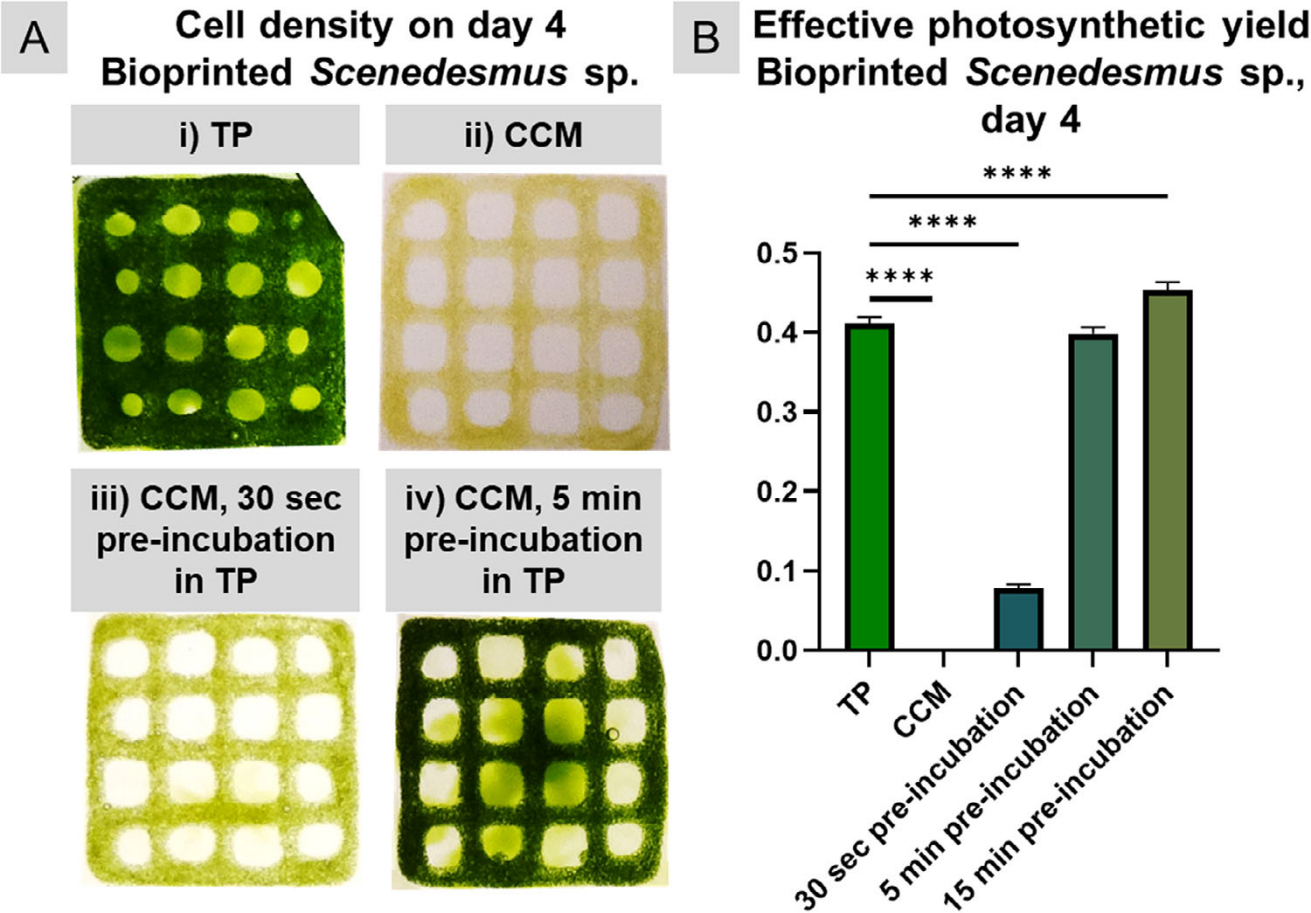
Effect of the co‐culture medium (CCM), without and with pre‐incubation in TP medium, on bioprinted Scenedesmus sp. (A) Visual comparison of bioprinted scaffolds after 4 days of cultivation under red light illumination in (i) in standard algal TP medium (control), (ii) in CCM, (iii, iv) in CCM after pre‐incubation in TP medium for 30 s and 5 min, respectively. (B) Effective photosynthetic yield as a measure of cell health and photosynthetic activity of microalgae after four days of cultivation in CCM, without and with pre‐incubation in TP medium, and in comparison to the standard algal TP medium (n = 5). Statistical significance: ^****^ *p* < 0.0001.

Although most components of the CCM were previously studied for their effect on *Scenedesmus* sp. and found to be compatible, the bioprinted microalgae initially did not survive cultivation in the final CCM. To combat this effect, the bioprinted microalgae constructs were immersed in TP medium directly after crosslinking before replacing it with CCM (“pre‐incubation step”). The impact of the CCM and the pre‐incubation procedure was not apparent immediately after printing, but became noticeable over the first few days of cultivation as shown in Figure 4A. Subfigure A (i) depicts the normal growth behavior of *Scenedesmus* sp. after bioprinting over four days of cultivation; (ii) illustrates the absence of growth in the scaffolds in the CCM; subfigures (iii) and (iv) show the positive effect of 30 s and 5 min of pre‐incubation on the immobilized microalgae.

As a measure of photosynthetic activity and cellular health of the microalgae, the effective photosynthetic yield was measured as described previously [29] and briefly in chapter 4.10. As shown in Figure 4B, no photosynthetic yield was detected after four days in samples that were incubated in CCM immediately after printing. However, pre‐incubation in TP medium of just 30 s improved the photosynthetic yield notably to 0.08, with a 5‐min pre‐incubation showing results comparable to the control group (Y(II) = 0.4 and Y(II) = 0.41, respectively). Separate experiments have verified that this short‐term pre‐incubation process does not adversely affect the INS‐1 cells or the islets (data not shown).

**FIGURE 5:**
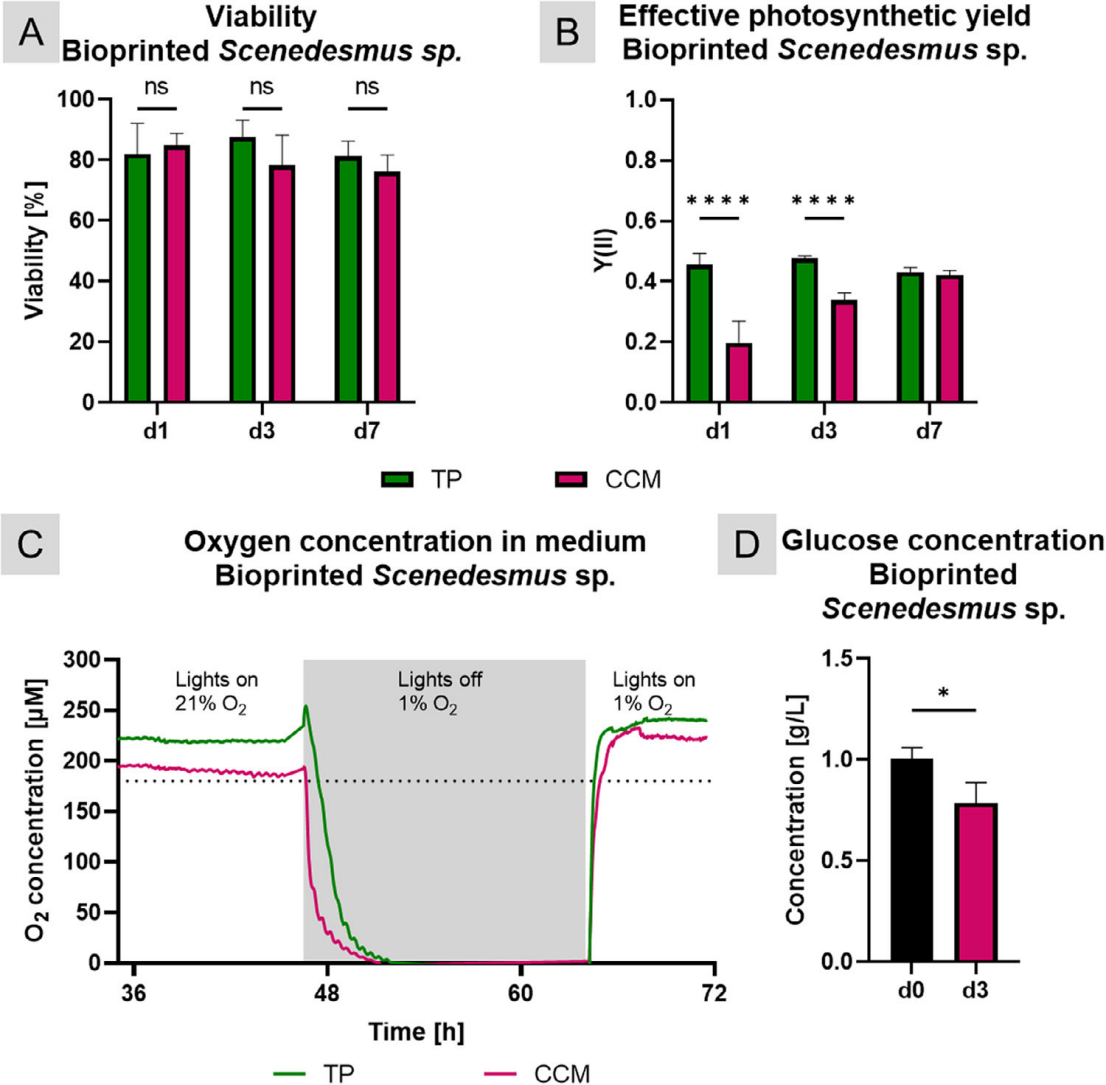
Influence of the co‐culture medium (CCM) on bioprinted Scenedesmus sp. After bioprinting, the scaffolds were pre‐incubated in TP medium for 5 min and then cultivated in CCM. (A) Viability (n = 6, mean±SD) and (B) photosynthetic efficiency (n = 10, mean±SD) over 7 days of cultivation under red light illumination; (C) oxygen production of the microalgae in three phases immediately after bioprinting and pre‐incubation in TP medium: 21% environmental O_2_ and red light illumination (“Lights on”), followed by a switch to hypoxic conditions (1% O_2_) and cultivation in darkness (“Lights off”) until total depletion of oxygen, before the illumination was resumed (“Lights on”) under hypoxic conditions (n = 4, mean), dotted line: theoretical normoxic conditions; (D) Glucose concentration in co‐culture medium before and after 3 days of cultivation under red light illumination (n = 4, mean±SD). Statistical significance: ns: not significant; ^*^ *p* < 0.05; ^****^ *p* < 0.0001.

Following the introduction of the 5‐min pre‐incubation period, the viability, photosynthetic yield, and oxygen production of Scenedesmus sp. in CCM were compared to those in standard TP medium. As illustrated in Figure 5A,B, the number of viable microalgae over seven days of cultivation was comparable in both media (representative images shown in Figure S2). However, the photosynthetic yield was initially significantly lower in the co‐culture medium but recovered to levels similar to the control on the seventh day, implying a necessary adaptation phase. Subsequently, the functionality of bioprinted Scenedesmus sp. in CCM vs. standard TP medium was evaluated by measuring the oxygen levels in the surrounding liquid across three phases: In phase 1, starting immediately after printing and crosslinking, the microalgae were cultivated under red light illumination in normoxic conditions (21% O2). In phase 2, the light was turned off, and the oxygen concentration in the incubator was reduced to 1% until complete depletion of oxygen in the media. In phase 3, the light was turned on again while maintaining a hypoxic environment to investigate the duration required for the microalgae to restore normoxic conditions in the two media via photosynthetic oxygen production.

As depicted in Figure 5C, even after the inclusion of the pre‐incubation step the O_2_ concentration was slightly reduced in the CCM in phase 1; however, oxygen levels above normoxic conditions were maintained in both media. During phase 2, the rate of oxygen depletion was faster in the CCM; the oxygen content fell to under 50 × 10^−6^ м after 60 min in CCM and, much more slowly, after 180 min in TP medium and finally reached 0 in both media. In phase 3, the restoration of normoxic conditions was already complete after 20 min in TP medium, whereas 50 min were needed in CCM; in both media, hyperoxic conditions were achieved. Notably, the oxygen content in CCM reached an overall higher level in phase 3 than in phase 1, but was still slightly reduced compared to TP.

In a previous study, we examined the effect of high glucose levels on the photosynthetic activity of *Scenedesmus* sp., observing that this microalga prefers autotrophy even when organic carbon sources are available [29]. To confirm this, glucose levels were measured before and after three days of cultivation in CCM and under red light illumination. Although glucose levels dropped significantly, sufficient glucose for the beta cells to survive remained.

In summary, the microalgae could be cultivated in the CCM, but a short pre‐incubation step in TP medium was essential. Even after the successful inclusion of this pre‐incubation, four days of cultivation were needed to adjust their metabolism to the non‐ideal medium and reach a comparable photosynthetic efficiency to the culture in TP medium. This can in part be explained by the reduced content of salts that make up most of the microalgae's nutrition. Nevertheless, even the reduced photosynthetic activity compared to the standard culture was sufficient to consistently maintain normoxic conditions from the beginning of the experiment, that is, from the start of culture in the incubator immediately following the 5‐min pre‐incubation.

Most microalgae have a mixotrophic metabolism, meaning they can metabolize organic carbon sources – if present – while using oxygen (heterotrophy) and also metabolize inorganic carbon sources, such as CO_2,_ during photosynthesis, while producing oxygen (autotrophy). In phase 1 of the experiment, the slightly reduced oxygen content in CCM compared to TP medium can be attributed to two factors: (1) reduced photosynthetic activity in microalgae in CCM before full adaptation and (2) a partial switch to heterotrophy. This switch is also evident in the reduced glucose content in CCM, as shown in Figure 5D. In phase 2, the reduction in oxygen indicates a shift to full heterotrophic metabolism in both media due to the absence of light. The decrease in CCM follows a steeper curve, presumably due to the availability of glucose in the medium and from internal carbohydrate storage. Meanwhile, microalgae in the TP medium could only metabolize glucose from their internal storage. Phase 3 shows nearly exclusive autotrophic metabolism and high oxygen production. Microalgae in both media restored normoxic conditions almost simultaneously. Microalgae cultivated in CCM reached higher oxygen levels in this phase than in phase 1, suggesting ongoing adaptation to the non‐optimal conditions as well as an increased cell number. This mixotrophic behavior may disrupt the growth and functionality of the mammalian partner. However, given the higher initial glucose content and more frequent medium changes in islet cultures, it is unlikely for the heterotrophic aspect of the microalgae to adversely affect the islets in co‐culture.

Since we could verify overall that no significant difference to the standard medium was detectable in viability or functionality of either INS‐1 cells or islets when using the CCM, we were able to find a balance between both media, which should allow a long‐term in vitro co‐cultivation.

In the literature, there are few examples of successful co‐cultures between mammalian cells and microalgae over several days, all utilizing the microalgae *Chlamydomonas reinhardtii* as a natural oxygen generator. Hopfner et al. [22] established a co‐culture with human fibroblasts and utilized a 1:1 mixture of Dulbecco's Modified Eagle's Medium (DMEM, for human cells) with 20% FBS (10% final concentration) and TRIS Acetate Phosphate medium (TAP, for microalgae), counteracting anoxic environmental conditions while maintaining growth and regular functions of both cell types over seven days. Maharjan et al. [38] used a similar CCM (1:1 mix of DMEM and TAP medium, with 10% FBS and 1% P/S) to establish co‐cultures with the carcinoma‐derived liver cell line HepG2. Later, the same group demonstrated optimal growth of C. reinhardtii and growth and differentiation of C2C12 muscle cells in a medium consisting of 3 parts DMEM and 1 part TAP [39].

Under hypoxia, the viability and functionality of the mammalian cells were enhanced in co‐culture, however, a harmful effect of the antibiotic supplement to the microalgae was noted, limiting cultivation to four days. In a previous study, we found similar adverse effects of streptomycin and consequently assessed the influence of antibiotic supplements on *Scenedesmus* sp. The semisynthetic antibiotic ampicillin was identified as an optimal replacement for the microalgae‐toxic penicillin/streptomycin combination [29].

### 2.6 Direct co‐culture as Proof‐of‐concept

After successfully determining the parameters necessary for co‐cultivation, we moved toward a direct co‐culture experiment. This proof‐of‐concept focused on maintaining the viability and function of both partners in a four‐day co‐culture, as a step toward long‐term in vitro cultivation. Islets of 10 female Wistar rats were isolated and pooled according to the protocol presented in the experimental section, yielding ∼5000 IEQ; the experiment was repeated once.

A ring of bioprinted *Scenedesmus* sp. was placed around a macroporous construct of bioprinted islets to avoid direct contact while maintaining short diffusion ways (Figure S1). The cultivation was conducted under red‐light illumination in hypoxic conditions (1% environmental oxygen), and the CCM was replaced after 2 days. After 4 days of cultivation, the medium was removed, the glucose‐stimulated insulin response was assessed under hypoxic conditions, and cell viability was evaluated using live/dead staining.

**FIGURE 6:**
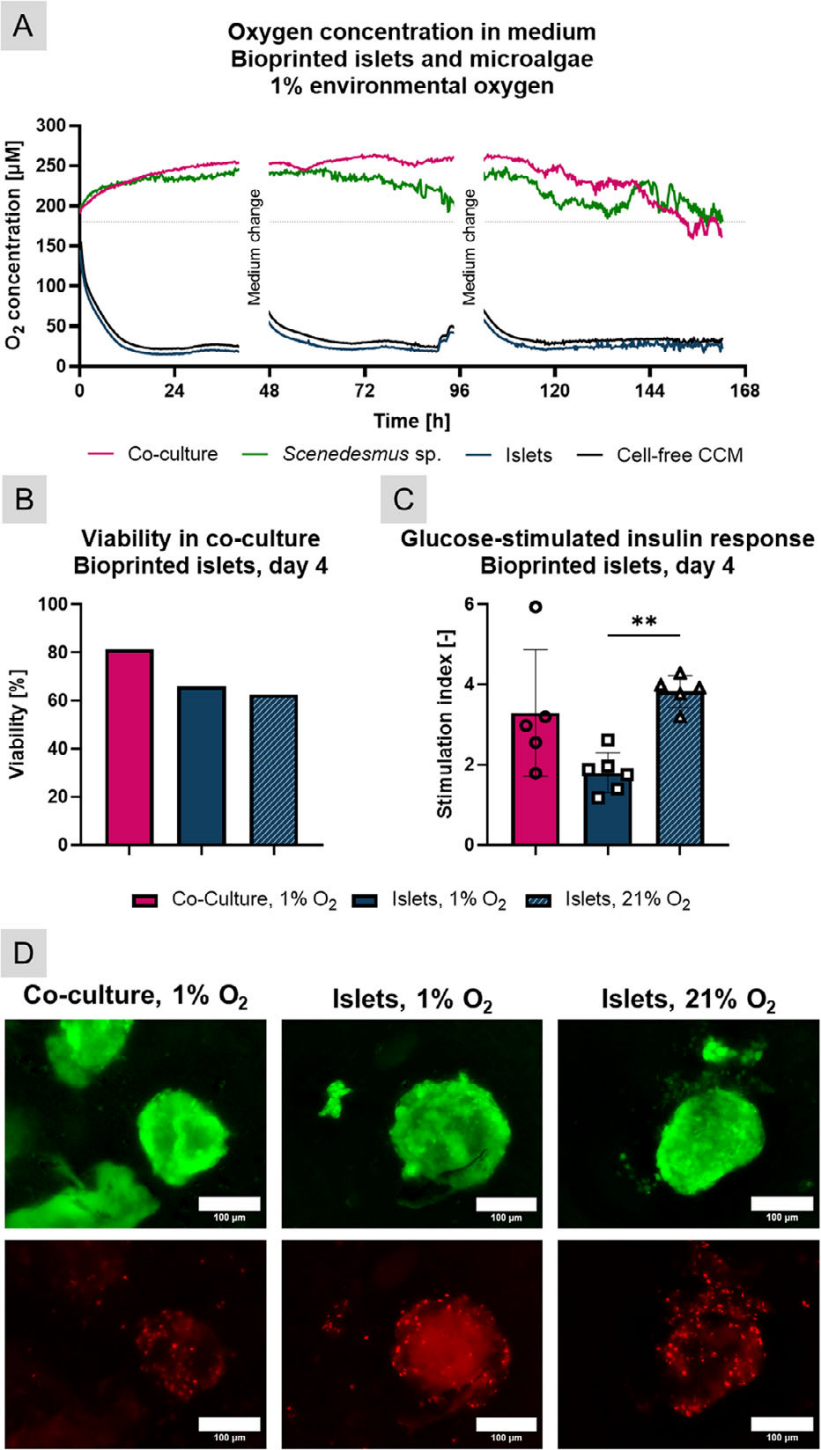
Co‐culture between bioprinted rat islets and bioprinted Scenedesmus sp. in hypoxic condition (1% O_2_), compared to bioprinted monocultures (“Islets”) in either hypoxic (1% O_2_) or normoxic (21% O_2_) conditions. Cultivation was performed in a co‐culture medium (CCM) under red‐light illumination. (A) Dissolved oxygen concentration in the medium over the course of the cultivation of bioprinted co‐cultures of islets and Scenedesmus sp. (“Co‐culture”) in hypoxic conditions, Scenedesmus sp. in bioprinted mono‐culture (“Scenedesmus sp”) served as positive control and islets in bioprinted mono‐culture (“Islets”) served as negative control; dotted line: theoretical normoxic O_2_ content (n≥4, mean); (B) Viability of islets in bioprinted co‐culture with Scenedesmus sp. in hypoxic conditions after 4 days of cultivation, bioprinted mono‐cultures of islets in hypoxic and normoxic conditions served as controls (n = 12, mean); (C) Glucose‐stimulated insulin response of islets in bioprinted co‐culture with Scenedesmus sp. in hypoxic conditions after 4 days of cultivation, bioprinted mono‐cultures of islets in hypoxic and normoxic conditions served as controls (n≥5); (D) representative images of live/dead staining of bioprinted islets under same conditions as in C and D (green: live cells, red: dead cells). Statistical significance: ^**^ *p* < 0.01.

As shown in Figure 6A, continuous oxygen measurement over the course of the experiment demonstrated that the microalgae maintained hyperoxic conditions in co‐culture for four days, with islets not impairing oxygen production. The viability of islets in co‐culture was 81%, which was higher than the normoxic islet control at 62.5%. The stimulation index was comparable between both groups, at 3.3 and 3.8, respectively. Islets cultivated in hypoxia had a similar level of viability to the normoxic group at 66% but showed a reduced response to glucose stimulation with an SI of 1.8 (Figure 6B,C). Subfigure D depicts representative fluorescence microscopy images of islets, showing a necrotic core in the hypoxic islet group. In contrast, the islets in co‐culture displayed no distinctly visible changes in viability compared to the normoxic group (see also Figure S4).

In summary, the data indicate that a coculture of microalgae in the vicinity of pancreatic islets in bioprinted hydrogel constructs can provide sufficient oxygen to maintain the viability and functionality of the islets. The observation that islets in the co‐culture even surpassed the survival rate of the normoxic control could be explained by the fact that the islets had a higher oxygen content available due to the small distance to the oxygen‐producing microalgae as well as an oxygen production rate that exceeded normoxic conditions. This could accelerate the diffusion of oxygen into the core of islets, preventing local necrosis. In the normoxic control, diffusion distances from the surface of the medium to the bioprinted construct are comparably long, which might result in hypoxic conditions in the microenvironment of the islets. Buchwald et al. [40] reported that only 10% oxygen was available to isolated islets under standard cultivation conditions (21% environmental oxygen, static culture), leading to hypoxia‐related necrosis. Therefore, it is possible that islets in in vitro cultures might require hyperoxic environmental conditions to guarantee sufficient oxygen tension and to maintain their metabolism and viability, as stated by Komatsu et al. [41, 42]. However, this hypothesis is not corroborated by the glucose‐stimulated insulin response results. No significant difference was observed between the SI of islets in the hyperoxic co‐culture and the normoxic control group, whereas the islets cultivated under hypoxic conditions exhibited the anticipated reduction in functionality.

The presented proof‐of‐concept was highly successful, since the here described “distance co‐culture” (not separated, but only very limited direct contact) with microalgae effectively compensated for environmental hypoxia by providing sufficient oxygen to achieve hyperoxic conditions while maintaining hydrogel integrity. As demonstrated by Bloch et al. [19], islets that were co‐incubated for a period of 3 h in direct contact with photosynthetically active microalgae exhibited a higher Stimulation Index (SI) under hypoxic conditions in comparison to islets cultivated alone. In this study, a similar positive outcome was observed for a multi‐day co‐culture, achieved through the optimization of illumination parameters and the development of a specialized co‐culture medium. Furthermore, it was observed that hyperoxia, also noted in this study, did not impair islet function. In contrast, hypoxia and anoxia were found to have a negative impact on islet function.

The number of microalgae needed to produce enough oxygen for the islets needs to be optimized to adequately support the islets but still minimize the microalgae cell number to in turn, minimize the amount of bioink needed. Bloch et al. [19] found that 11 000 cells of the microalgae *Chlorella sorokiniana* under optimal illumination were necessary to produce oxygen for one islet. When cultivating both cell types separately, the number of algae had to be increased significantly [20]. Using cyanobacteria as oxygen generator, Evron et al. [21] found that 300 µg chlorophyll was sufficient to support 1200 IEQ, but here the separate encapsulation and the resulting long diffusion ways need to be taken into account.

In our study, we calculated that approx 11 000 *Scenedesmus* sp. cells were utilized in the co‐culture. The oxygen levels in the co‐culture matched or slightly exceeded those in the microalgae monoculture, indicating a balance between the net oxygen production rate of *Scenedesmus* sp. and the oxygen consumption rate of the islets. This suggests that the ratio between microalgae and islets per construct under 100 µmol m^−^^2^s^−1^ illumination was adequate. According to the literature, 1200 islets are sufficient to restore blood glucose control in a diabetic rat [21]. In the experiments described here, approximately 440 IEQ were printed into one construct. Increasing the cell concentration and enlarging the construct slightly (from ∼1 cm^2^ to 1.5 cm^2^) for an in vivo study is possible; tripling the number of *Scenedesmus* sp. can also be accomplished in the current design by increasing the number of layers.

### 2.7 Spatiotemporal Oxygen Distribution

The measurement of dissolved oxygen in the medium in combination with the observed improvement of islet health, proved that microalgae can counteract hypoxia within the described co‐culture system as a whole. To gain insights into the spatiotemporal oxygen distribution within the bioprinted construct, luminescent optical sensor nanoparticles (SNP) are a great tool to visualize possible hypoxic zones and to optimize the co‐culture design. Incorporating SNP into hydrogels laden with INS‐1 and microalgae enabled the online imaging of oxygen concentration and to map the heterogenous oxygen distribution within the scaffold. Since the biocompatibility of the SNP and pancreatic islets was not investigated previously, the model cell line INS‐1 was chosen here due to their similarly high metabolic activity and oxygen consumption and to minimize the use of primary cells. Materials, preparation, and functionality of these SNP are described elsewhere [26, 43, 44]. The SNP contains an inert fluorescent dye (Bu3Coum) with constant emission of green light, and an O_2_‐sensitive luminescent indicator (platinum(II) *meso*(2,3,4,5,6‐pentafluoro)phenyl porphyrin) emitting red light when excited by blue light. This allows for a ratiometric measurement of oxygen distributions within bioprinted, cell‐containing constructs. By using an SLR‐based camera system, multiple constructs can be imaged simultaneously.

**FIGURE 7:**
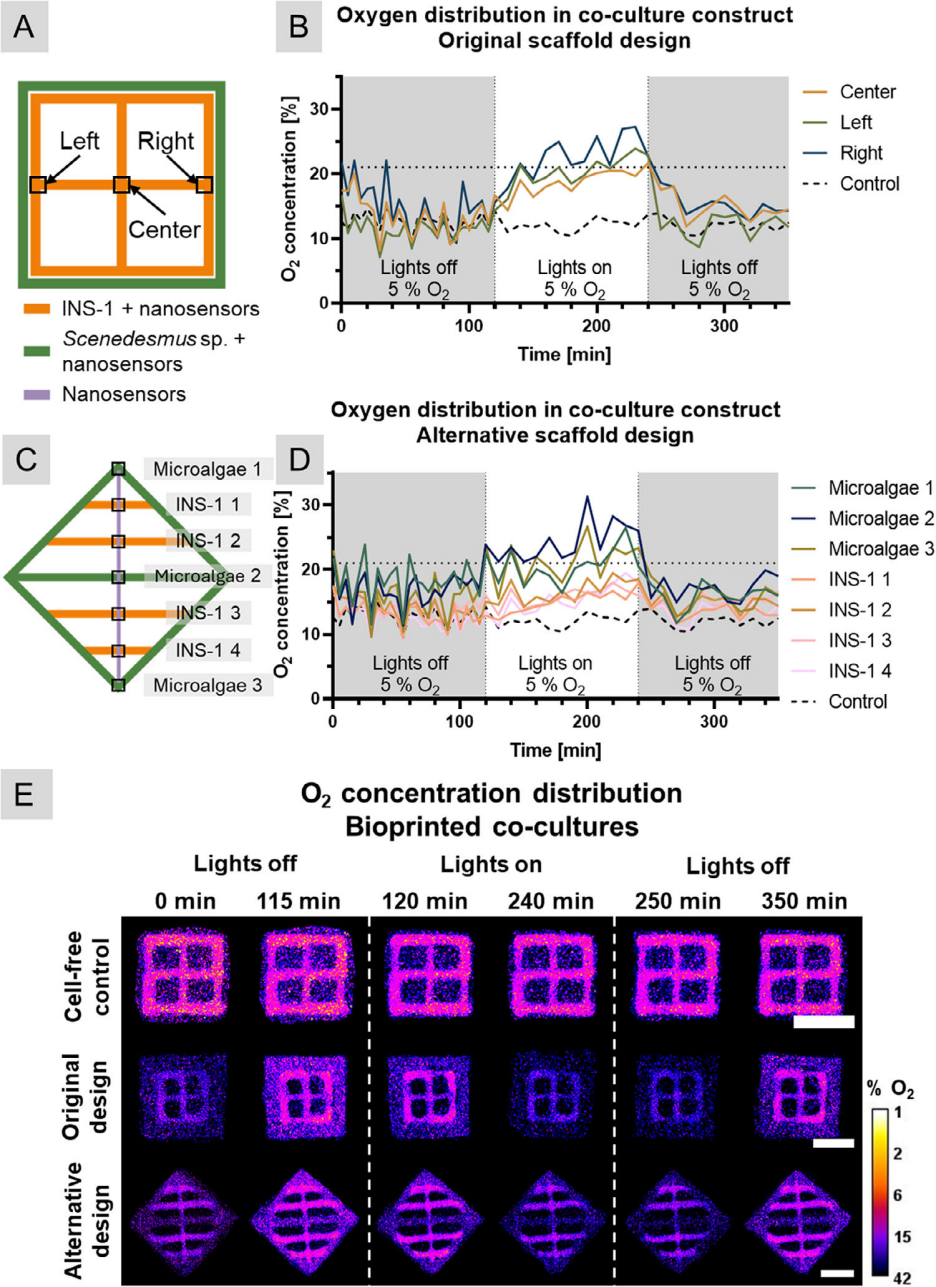
Spatiotemporal oxygen distribution in bioprinted co‐cultures of INS‐1 cells and Scenedesmus sp. under hypoxic conditions after four days of cultivation in co‐culture medium (CCM). (A) Sketch of the construct design used throughout the study, and (C) sketch of the suggested alternative design, areas analyzed for oxygen dynamics are marked. (B, D) oxygen concentration within the marked areas over time in 3 phases: (1) without illumination; (2) under red‐light illumination; (3) again without illumination. (E) Images of the O_2_ concentration distribution over the bioprinted co‐cultures at the start and end of each respective phase. Calibration bar: O_2_ concentration measured by nanoparticles in images; scale bar: 5 mm.

Distance co‐culture constructs as described above (“original scaffold design”) were investigated regarding the presence of possible hypoxic zones. Additionally, an alternative scaffold design was printed and analyzed in the same way; here, strands of INS‐1 and *Scenedesmus* sp. were placed alternating in one layer to avoid long distances between microalgae strands and potentially allowing for a potentially more homogenous oxygen distribution. Sketches of both designs are depicted in Figure 7A,C.

For this experiment, a co‐culture of INS‐1 and *Scenedesmus* sp. was utilized. As outlined in Chapter 4.4, bioinks containing INS‐1 or *Scenedesmus* sp. were functionalized with SNP prior to printing. In the case of the alternative scaffold design, a strand of cell‐free ink containing an SNP was printed perpendicular to the other strands, with the objective of obtaining more information about the oxygen distribution differences between the strands. The bioprinted cell constructs were cultivated under red‐light illumination in CCM in normoxic conditions for a period of three days prior to analysis, thus allowing the microalgae to acclimatize to the medium and achieve peak functionality. Then, the environmental oxygen level was reduced to 5%, and the constructs were cultivated overnight under illumination until depletion of oxygen in the cell‐free control. On day 4, an experiment in three phases was carried out: (1) O_2_ levels in the dark were measured every 5 min for 2 h; (2) red‐light illumination was turned on for 2 h and sensor measurements were taken every 30 min during this period, as the light had to be turned off briefly for this purpose; and (3) the lights were turned off again and oxygen depletion was investigated. Three samples of each group were cultivated: original design, alternative design, and cell‐ and SNP‐free control. The results of one representative sample of each group are shown here.

Figure 7B shows the oxygen levels within the INS‐1 strands for the original designs. The “middle” position is the farthest from the microalgae ring, while the “left” and “right” positions are next to a microalgae strand. During the initial dark phase, oxygen levels decreased to similar hypoxic levels for all three positions. Under illumination, a significant increase in oxygen concentration toward normoxic conditions was evident for all three positions, though the center exhibited slightly lower levels. At the start of phase 3, a rapid decrease in oxygen was visible for all three positions. Interestingly, even though the incubator was set to 5% environmental oxygen, the O_2_ levels only reached a minimum of approximately 12%. This is likely due to the setup of the measurement system. The camera was placed below the samples, which are positioned high in the incubator, close to the fan and wall openings that allow cables to pass into the incubator. This brings oxygen‐rich air from outside in close proximity to the samples in turbulent air, leading to a higher oxygen concentration. Nevertheless, the cell‐free control group maintained the same oxygen concentration throughout the experiment, indicating a successful co‐culture with oxygen production. The oxygen concentration reached 25% and 23%, respectively, in both outer positions, while 21% was achieved in the middle of the construct. These discrepancies are likely due to the much shorter diffusion distances between the outer positions of the INS‐1 construct and the microalgae ring. Nonetheless, normoxic conditions were restored in all positions.

Figure 7D shows the nanosensor results for the alternative design. Here, three microalgae‐laden strands and four INS‐1‐laden strands were monitored over the same dark‐light‐dark phases. During the dark phases, hypoxic conditions similar to those of the original design constructs were encountered. During the light phase, the microalgae‐laden strands achieved hyperoxic levels ranging from 23% to 30% oxygen. Interestingly, despite the smaller maximum distance between the INS‐1 and microalgae strands compared to the original design, normoxic conditions could not be restored within the INS‐1 strands.

Figure 7E shows the images of the oxygen concentration distribution over the analyzed constructs at six different time points: (1) at the beginning of the measurement (0 min), (2) at the last measurement point of phase 1 in the dark (115 min), (3) at the first measurement point of phase 2 under illumination (120 min), (4) at the last measurement point under illumination (240 min), (5) at the first measurement point of phase 3 without illumination (250 min), and (6) at the last measurement point of the experiment after 2 h in the dark (350 min). Due to the nature of the measurement, a stronger color signal indicates a lower oxygen concentration. The cell‐free control shows hypoxic conditions throughout the construct and throughout the experiment, as seen in the O_2_ concentration diagrams. For the construct in the original design, the microalgae strands contained more oxygen at the beginning of phase 1 (dark), and oxygen depletion in the INS‐1 strands was visible. Between the start and end of phase 1, the INS‐1 consumed most of the available oxygen. Interestingly, the microalgae‐laden strands still exhibited a higher oxygen concentration, indicating slower oxygen consumption. This finding validates the hypothesis that *Scenedesmus* sp. prefers autotrophic metabolism even in the presence of glucose. Oxygen concentration increased after the onset of illumination in phase 2, achieving normoxia throughout the INS‐1 scaffold. After 2 h in darkness in phase 3, the O_2_ concentration in the INS‐1 strands was much lower than in the microalgae strands, indicating high cellular activity. The alternative design construct revealed that hypoxic conditions in the INS‐1 strand were never fully alleviated, despite the microalgae‐laden strands exhibiting similar oxygen levels to those in the original design.

In a previous study, a comparable experiment was performed involving co‐cultures of human mesenchymal stem cells and the microalgae *Chlorella sorokiniana* [26]. In this investigation, shorter periods of darkness and illumination were examined in a construct similar to the “alternative design” described here. Interestingly, the microalgae strain used in that study could restore normoxic conditions during illumination, but it used up the available oxygen much faster in the dark than the human cells did. This indicated a strong heterotrophic component of this microalga's metabolism, which was proven in a later study [29].

In conclusion, spatiotemporal analysis of oxygen concentration within the co‐culture constructs showed that normoxic conditions were restored throughout, enabling all immobilized mammalian cells to grow and function properly. While the proposed alternative design did not offer an improved oxygen supply, these results can be used as a first step toward optimizing the design of the bioprinted co‐culture system.

### 2.8 Contribution to the Field and Limitations of the Study

The co‐culture system described in this study is the first to systematically investigate and optimize the impact of co‐culture conditions on both involved cell types—rat islets and photosynthetic microalgae—rather than focusing solely on the requirements of the mammalian cell partner. An important innovation is the identification of red light as the optimal illumination source for the co‐culture. Red light did not negatively affect the viability or function of bioprinted INS‐1 cells or primary islets, making it suitable for supporting photosynthetic activity in microalgae without compromising the viability or function of the mammalian cells. This finding is particularly relevant given the sensitivity of islets to phototoxicity and the need to balance light requirements for photosynthesis with preserving islet function [32, 45]. Both the developed co‐culture medium and the illumination are important for enabling the potential development of long‐term co‐cultures, as the viability and function of both cell types are essential for the success of such systems in future therapeutic applications. The same principle applies to co‐culture systems involving other mammalian cells.

The microalgae partner predominantly used for co‐cultures in the literature is *Chlamydomonas reinhardtii* [22, 46], though *Synechococcus* cyanobacteria have also been used in some studies [21, 47]. In the scope of this study, *Scenedesmus* sp. was presented as a new possible microalgae partner that is similarly well investigated to *C. reinhardtii*. However, it might be better suited to long‐term investigation under co‐culture conditions due to its higher tolerance for temperatures of up to 37°C without reduced growth rates [48] and continuous illumination [49]. Furthermore, *Scenedesmus* sp. adapts well to red‐light illumination and immobilization, and maintains a predominantly autotrophic metabolism in the presence of glucose, in contrast to *C. sorokiniana* [29].

Initial experiments using the INS‐1 cell line provided the first information about the necessary components of the medium and the influence of light. This strategy was cost‐effective and easy on resources for early‐stage optimization and reduced the use of animals, aligning with the principles of the 3Rs (Replacement, Reduction, Refinement) in animal research. However, the applicability of the INS‐1 as a model cell line was found to be limited: Inexplicable variations between samples and experiments rendered the comparison of results difficult and led to the decision to focus solely on the islets for the final direct co‐culture experiments.

Another major contribution is the development of a co‐culture medium that supports the survival and functionality of both islets and microalgae – to the authors’ best knowledge no study to date has made a similarly comprehensive attempt. The formulation of this medium required careful optimization of carbon sources and supplements to meet the metabolic needs of both cell types. The resulting medium enabled reliable and reproducible co‐culture experiments, providing a robust platform for further investigation and optimization. Importantly, the protocol established for identifying essential medium components and their ratios can be adapted for other co‐culture systems involving mammalian cells and microorganisms, broadening the impact of this work beyond the specific context of islet‐microalgae co‐culture.

Despite these advances, several limitations must be acknowledged. First, the co‐culture medium developed for in vitro experiments is not directly applicable in an in vivo context, where nutrients and fluids are supplied by the host organism. Nevertheless, this medium development is necessary for prior in vitro investigations to optimize and characterize the co‐culture system.

A further limitation regards the functionality of the islets in in vitro culture. A previous study [27] has shown that islets present a pronounced reduction in glucose‐stimulated insulin secretion after one week of in vitro culture; therefore, the cultivation time was limited to four days in this study. Additionally, the use of older donor rats in the present study might have contributed to the comparatively lower islet viability and SI, since the advanced age of the rats could impact the functionality of the isolated islets. Both these factors hindered the development of a genuine long‐term in vitro co‐culture. Furthermore, the number of biological replicates, including pancreatic islets, was kept to a minimum for ethical reasons. Although repetition experiments with younger animals might have yielded a higher SI and less statistical variance, reliable conclusions could be drawn from the provided results and preliminary experiments involving INS‐1 cells. Subsequent in vivo studies may be able to provide reliable conclusions regarding the long‐term applicability of the co‐culture system [17, 21].

The co‐culture system also faced challenges related to direct cell‐cell interactions. Preliminary experiments revealed that direct contact between islets or INS‐1 cells and microalgae negatively impacted microalgae survival. Drawing from the literature, Evron et al. [21] achieved a stable production of oxygen that supported the function of islets in vivo for seven days by separating the oxygen generator (in this case the cyanobacteria *Synechococcus lividus*) physically from islets using a gas‐permeable membrane. Thus, a long‐term culture was possible that could provide oxygen for one month, avoiding the challenges associated with a direct co‐culture, such as light influence, different medium requirements, and inhibitory cell interactions. However, this two‐compartment approach is not ideal for oxygen diffusion, as physical separation can limit the efficient transfer of photosynthetically produced oxygen to the islets. To avoid the long diffusion pathways, 3D bioprinting was implemented as a fabrication method in our work. This approach allows for the adaptable design of a macroporous construct, ensuring the incorporation of both cell types within a single construct. This facilitates short diffusion distances with minimal direct contact between microalgae and islet‐laden strands. The current scaffold design used in the present study, while functional, represents a compromise between cell survival and oxygen delivery. In future studies, the underlying causes of this negative influence of the mammalian cells on the microalgae, as well as potential solutions, need to be evaluated. Optimal scaffold designs must be developed, based especially on the findings of the spatiotemporal oxygen distribution measurements, and here a more elaborate combination of functional monitoring and numerical modeling could enable such optimization [50].

### 2.9 Outlook

To meet the insulin requirements of a diabetic human patient, approximately 500 000 islets are needed [21]. This necessitates a significant increase in both the islet density and the construct size, along with the required number of microalgae: based on the ratio between microalgae and islets applied in this study, approximately 5.5 × 10^9^ Scenedesmus sp. cells would be required to support the islets. To determine the optimal ratio between both cell types, developing a mathematical model that describes oxygen production of the algae depending on the locally available light energy, diffusion characteristics of the bioink, and oxygen consumption of the pancreatic islets is necessary. Taking all these parameters into account, the exact cell ratio required for this system can be calculated and adapted. Achieving this could involve increasing the cell concentration in the bioink; however, even with a 20‐fold increase, 55 g of microalgae‐laden bioink would be required per construct. Consequently, to supply oxygen for a bioartificial pancreas for a human patient, upscaling and optimization in design, illumination, and photosynthetic efficiency are essential.

Regarding the potential for in vivo application, it is necessary to address how the developed co‐culture system can be translated into an implantable device. Such insulin‐producing devices, as developed by Barkai et al. (2013) and Ludwig et al. (2013), have been successfully implanted in rats and even in a diabetic patient [16, 17]. Inspired by these designs, the co‐culture system described here should also be enclosed within a plastic housing containing a polytetrafluoroethylene membrane. This membrane would permit nutrient diffusion while preventing immunological interactions. This approach provides mechanical protection and enables the integration of a necessary light source, such as an OLED sheet attached to the interior of the housing. Additionally, it acts as a safety measure to prevent microalgae from entering the recipient's body, addressing the need to prevent microalgae contamination.

Finally, potential long‐term negative effects of co‐encapsulating islets and microalgae must be considered. Overgrowth of microalgae and a metabolic shift from photosynthesis to heterotrophy could lead to competition for nutrients and compromise islet function. Possible solutions include implementing a dark/light cycle of illumination or reverting to the approach of separating the cell types utilizing gas‐permeable membranes and trying to optimize the gas flow in such a system. To verify islet health in co‐culture, markers for oxidative stress and hypoxia should be analyzed as well as pro‐inflammatory cytokines to exclude that algae trigger an inflammatory response in the islets. Additional quantitative metabolic assays could give a deeper understanding of islet viability.

Looking forward, several strategies for improvement and further research were identified. First, the scaffold design needs to be further optimized to enhance oxygen diffusion while minimizing detrimental direct contact between islets and microalgae. To address the scalability challenge, future work should focus on increasing the photosynthetic efficiency and oxygen production of microalgae. This could be achieved through genetic or metabolic engineering, optimizing light management, and improving cultivation systems to maximize light utilization and minimize self‐shading.

## 3 Conclusions

In this study, we developed and optimized a co‐culture system composed of pancreatic islets and photosynthetically active microalgae, specifically *Scenedesmus* sp., bioprinted in alginate‐based hydrogels. The main findings highlight the careful balance required to support the viability and function of both mammalian and microalgal partners. One key factor was the identification of red light as an optimal illumination source, which maintained photosynthetic activity in microalgae without detrimental effects on islet or INS‐1 cell viability and function. Most importantly, a co‐culture medium was formulated to meet the metabolic requirements of both cell types; a brief pre‐incubation of microalgae in their standard medium prior to co‐culture was essential for their adaptation and photosynthetic efficiency. Direct co‐culture experiments under hypoxic conditions demonstrated that microalgae‐generated oxygen maintained hyperoxic conditions in the medium as well as normoxic conditions directly within the construct, resulting in higher islet viability compared to normoxic controls and preserved glucose‐stimulated insulin secretion. These results establish that a spatially organized co‐culture in bioprinted constructs can effectively compensate for hypoxic stress, supporting both cell types over several days. Overall, this study developed a functional mammalian–microalgae co‐culture, with systematic optimization of light, medium, and spatial arrangement, demonstrating reliable oxygen delivery and preservation of islet function in vitro.

## 4 Experimental Section

### 4.1 INS‐1 Cell Culture

The rat insulinoma‐derived beta‐cell line INS‐1 was a kind donation from Prof. emeritus Claes B. Wollheim (MD Department of Cell Physiology and Metabolism, University Medical Center 1, Geneva, Switzerland) [30]. They were expanded in monolayer culture in T175 cell culture flasks (Sarstedt, Nümbrecht, Germany) at 37°C and 5 % CO_2_ in a HERAcell CO_2_ incubator (Thermo Fisher Scientific, USA) with twice‐weekly passaging. INS‐1 were cultivated in RPMI‐1640 medium with 11.1 mmol L^−1^ D‐glucose (Gibco, Life Technologies, USA) supplemented with 10 % heat‐inactivated fetal bovine serum (HI‐FBS, Gibco), 100 U mL^−1^ penicillin and 100 µg mL^−1^ streptomycin (P/S; both Gibco), 10 mmol L^−1^ HEPES pH 7.4 (Carl Roth Germany), 2 mmol L^−1^ L‐glutamine (Merck Millipore, Biochrom, Germany), 1 mmol L^−1^ sodium pyruvate (AppliChem, Germany), and 50 µmol L^−1^ 2‐mercaptoethanol (Sigma‐Aldrich, Germany).

### 4.2 Islet Isolation and Culture

Isolation of pancreata from rats was performed according to guidelines established by the Technische Universität Dresden Institutional Animal Care and Use Committee and was approved by the Commission for Animal Studies at the District Government Dresden, Germany (DD24.1‐5131/451/2 and DD25‐5131/554/5). Pancreata were obtained from wild‐type female Wistar rats at 10–22 weeks of age and digested with collagenase as described by Barkai et al. [17] with slight modifications. For each experiment performed in this study, islets from 8–12 rats were pooled. Briefly, the pancreata were infused with 10 mL of glucose‐free RPMI 1640 medium without supplements (Gibco) containing 1 mg mL^−1^ collagenase (Sigma, USA) and 1 µL mL^−1^ DNase (Roche, Switzerland) during the dissection and kept on ice until digestion at 37°C for 14 min. Digestion was stopped by the addition of ice‐cold wash buffer consisting of RPMI 1640, containing 5.5 mmol L^−1^ glucose, supplemented with 10% HI‐FBS. The tissue was homogenized, and islets were purified by centrifugation with a discontinuous Ficoll‐gradient (densities 1.125 g/1.096 g/1.08 g/1.069 g cm^−3^ in Euro Collins Solution, Sigma) for 15 min at 1590 g and 4°C. Purified islets were washed twice and kept in culture medium consisting of RPMI 1640, 5.5 mmol L^−1^ glucose, supplemented with 10% HI‐FBS, 10 mmol L^−1^ HEPES pH 7.4, and P/S (same concentration as stated in chapter 4.1) overnight under cell culture conditions (37°C, 5% CO_2_). Exocrine debris was then removed manually; islet equivalents (IEQ) were determined using dithizone staining, and islets were used for 3D bioprinting immediately afterwards.

### 4.3 Microalgae Cell Culture

*Scenedesmus* sp. CCALA 1074 was obtained from the Culture Collection of Autotrophic Organisms (CCALA) in Třeboň, Czech Republic, and inoculated in 50 mL modified TRIS‐phosphate medium (TP medium, according to [51]) with 0.75 g L^−1^ sodium nitrate replacing the ammonium chloride [29].

Cell expansion was performed in a Multitron incubator (Infors HT, Switzerland) at 37°C under constant white LED light at 30% intensity. 50 mL cell suspension was cultivated in 250 mL Erlenmeyer shake flasks (ROTILABO, Carl Roth) for 14 days, then 5 mL of culture was sub‐cultivated into 45 mL fresh TP medium.

### 4.4 Preparation and 3D Bioprinting of Bioinks

Extrusion‐based 3D bioprinting and preparation of bioinks were performed as described previously [25, 27, 29]. Briefly, a hydrogel blend of 3 g L^−1^ alginic acid sodium salt from brown algae (alg) and 9 g L^−1^ methylcellulose (MC, molecular weight ≈ 88 kDa, Sigma–Aldrich) was prepared. Research‐grade alginate (M/G ratio 1:2, Sigma–Aldrich) was used for bioprinting of INS‐1 cells and *Scenedesmus* sp. in monoculture, while clinical‐grade alginate from NovaMatrix (Pronova Up MVM, NovaMatrix, Norway) was used for bioprinting of the islets and *Scenedesmus* sp. in islet co‐culture. Alginate and MC powder were sterilized by autoclaving for 20 min at 121°C. On the day of bioprinting, alginate was dissolved in phosphate‐buffered saline (PBS, Gibco) in the case of islets and INS‐1 or in deionized water for the microalgae. Then, the powdered MC was added to the alginate solution and homogenized through rigorous stirring before letting the material rest for 90 min to allow swelling of the MC. A total of 2 × 10^7^ INS‐1 cells or 4000–5000 IEQ were suspended in 100 µL cell culture medium per gram of material and then mixed into and homogeneously distributed in the material through gentle stirring with a spatula. For *Scenedesmus* sp., 5 × 10^6^ cells were suspended in 100 µL TP medium per gram material and mixed using a static mixer according to [52]. For the spatiotemporal oxygen measurement, luminescent styrene maleic anhydride copolymer nanoparticles were added to the AlgMC hydrogels. Equal amounts of a 6 g L^−1^ alginate solution and a 5 mg mL^−1^ stock solution of the sensor nanoparticles were mixed before the addition of MC and cells, as described previously by Trampe et al. [26]. The resulting bioink was transferred into a 10 mL plotting cartridge (Nordson EFD, Germany) and plotted using a pneumatic‐driven extrusion printer (Bioscaffolder 3.1; GeSiM, Germany). The bioinks were extruded through dosing needles and crosslinked ionically for 10 min directly after printing by immersing the constructs in 70 mM SrCl_2_ solution. For mono‐cultures of INS‐1 and *Scenedesmus* sp., macroporous constructs with a base area of 16×16 mm and five strands per layer were fabricated using a 410 µm diameter dosing needle. To ensure the structural integrity of the islets, they were printed using a dosing needle with an 860 µm nozzle diameter. Here, constructs had a base area of 12 × 12 mm and consisted of three strands per layer. To avoid direct contact between microalgae and mammalian cell‐laden constructs in case of co‐cultures, the microalgae were printed in a 16 × 16 mm ring around the mammalian cells that were printed into 12 × 12 mm constructs.

### 4.5 Experimental Setup

Cultivation took place in a HERAcell CO_2_ incubator with adjustable environmental oxygen content (Thermo Fisher Scientific, USA) under standard cell culture conditions (37°C, 5% CO_2_). For all experiments, except for O_2_ measurements, samples were placed in 12‐well plates and submerged in 1 mL of their respective cell culture medium.

Illumination was provided by a water‐ and heat‐resistant LED strip (LLV‐shop, Germany), placed 80 mm below the samples and providing homogenous red or white illumination at a photon irradiance of 80 µmol m^−2^ s^−1^ (2 mW cm^−2^). Light spectra for both illumination sources were recorded and published previously [34].

### 4.6 Assessment of Cell Viability

The cell viability of mammalian cells was evaluated using live/dead staining with calcein‐AM/ethidium homodimer‐1 (LIVE/DEAD Viability/Cytotoxicity Kit for mammalian cells, Thermo Fisher Scientific, USA) according to the manufacturer's protocol. Fluorescence microscopy with a BZ‐X800 fluorescent light microscope (Keyence, Japan) and subsequent semi‐automatic area determination of living and dead cells using ImageJ (Fiji, Version 1.52p) [53] were used to quantify cell viability of INS‐1. The viability of bioprinted microalgae was assessed by staining dead cells with SYTOX Green Nucleic Acid Stain (Thermo Fisher Scientific) and utilizing chlorophyll autofluorescence to visualize live cells as described previously [25]. Viability of Scenedesmus sp. and INS‐1 was calculated as the ratio of the area of living cells to the total area of both living and dead cells. For the semiquantitative assessment of islet viability, ∼10 islets of each experiment were imaged and then visually sorted into viability categories (0%, 25%, 50%, 75%, and 100%) before viability was calculated as describedusly [35].

### 4.7 Glucose‐stimulated Insulin Response

The analysis of insulin reaction in response to stimulation with glucose was done via glucose‐stimulated insulin response (GSIR) assay as described previously [27]. Whole scaffolds were used as single samples. For the free islet control, 20 islets of varying sizes were picked manually for each sample; the free control was used to confirm overall islet functionality for each isolation. On day 4 after bioprinting, scaffolds and free control islets were treated with low (3.3 mmol L^−1^) or high (16.4 mmol L^−1^) glucose in Krebs Ringer bicarbonate buffer (137 mmol L^−1^ NaCl, 4.7 mmol L^−1^ KCl, 1.2 mmol L^−1^ KH_2_PO_4_, 1.2 mmol L^−1^ MgSO_4_, 2.5 mmol L^−1^ CaCl_2_, 25 mmol L^−1^ NaHCO_3_, 0.25% BSA) and secreted insulin was quantified. First, all samples were exposed to 3.3 mmol L^−1^ glucose (resting conditions). For stimulation, samples were first treated with 3.3 mmol L^−1^ glucose, the supernatant was then collected, and samples were stimulated again with 16.4 mmol L^−1^ glucose. In addition, islets in chapter 2.2 and 2.3 were stimulated in low–high–low glucose conditions to check for a repeated adaptation to changing glucose levels (results available upon request). Supernatants were taken and stored at −20°C until the quantification of released insulin via high‐range rat insulin ELISA kit (Mercodia) was performed according to the manufacturer's instructions. The Stimulation Index (SI) was calculated by dividing the amount of insulin released in high glucose stimulation by the amount released in low glucose. The stimulation times varied between INS‐1 and islets; the former were placed in resting conditions for 30 min before low−high glucose stimulation was performed for 60 min each. The islets remained in resting conditions for 2 h, and stimulation was carried out for 3 h per glucose condition.

### 4.8 Oxygen Measurements

The optical oxygen measurement device “Resipher” (Lucid Scientific, Atlanta, GA, USA) was used for the oxygen concentration measurements in the medium. Bioprinted constructs were cultivated in a custom‐made, transparent 18‐well plate, in which it was possible to measure the dissolved oxygen content in six of the 18 wells. In each well, constructs were submerged in 1.5 mL medium while the oxygen content was measured continuously in the medium over the course of the experiment. Oxygen measurements were stopped prior to the GSIR assay due to the necessary frequent supernatant changes and the resulting oxygen fluctuation.

The O_2_‐sensitive sensor nanoparticles were prepared according to Mistlberger et al. [54]. The particles (both O_2_ indicator and reference dye) can be excited by blue light (within a spectral range of 400–475 nm) and emit an O_2_‐dependent luminescence in the red spectral region (625–720 nm with a distinct peak at 650 nm) along with an O_2_‐independent fluorescence from the reference dye in the green spectral region (475–550 nm). Bioprinted constructs were cultivated in standard 12‐well plates (Corning, USA), and ratiometric imaging of luminescence intensity was carried out from below the samples to avoid image distortion due to condensation on the lid. O_2_ imaging was performed using a ratiometric camera system [55] with a digital single‐lens reflex camera (EOS 1000D, Canon, Japan) mounted with a macro‐objective (Macro 100 f2.8 D, Tokina, Japan) and a 530 nm long‐pass filter (Uqgoptics, UK). A custom‐made blue LED lamp (445 nm, with a bandpass filter) was used to excite the O_2_‐sensitive nanoparticles. The camera and LED lamp were placed inside the cell culture incubator and controlled by the image acquisition software “look@RGB” (http://imaging.fish‐n‐chips.de). The software allowed the LED lamp to be triggered and images to be acquired at specific time intervals.

### 4.9 Glucose Measurements

Using 3,5‐Dinitrosalicylic acid (DNS), the glucose concentration of the supernatant was determined as a reducing sugar using a colorimetric process. 100 µL sample volume was mixed in a ratio of 1:1 with DNS‐reagent (DNS: D0550, Sigma‐Aldrich, and potassium sodium tartrate tetrahydrate: 131729.1210, Applichem) and heated in a water bath to 99°C. After diluting the sample with double‐distilled water in a ratio of 1:4, the extinction of the solution was analyzed using a microplate reader (Infinite M200 pro, Tecan) at a wavelength of 540 nm.

### 4.10 Photosynthetic Efficiency

The photosynthetic efficiency of microalgae was determined using variable chlorophyll fluorescence measurement on a pulse‐amplitude‐modulated (PAM) fluorometer utilizing the stationary measurement device MINI‐PAM blue (Heinz Walz, Germany, equipped with a blue LED), using the saturation pulse technique as described previously [29]. Briefly, to obtain information about the photosynthetic efficiency of *Scenedesmus sp*., the effective quantum yield of Photosystem II was recorded. Induction kinetics were recorded by applying repeated saturation light pulses to the illuminated samples in 20 s intervals over a span of 180 s (actinic light irradiance: 23 PAR). The effective quantum yield of Photosystem II in the algae, Y(II), was determined as Y(II) = (Fm’‐F)/Fm’, where F is the fluorescence yield in actinic light, and F’_m_ is the fluorescence yield during the saturation pulse. A high yield indicates a high capacity to channel absorbed photon energy into the microalgae for photosynthesis. To account for the macroporous structure of bioprinted samples, five Areas of Interest were specified on strands and crossings of the constructs and averaged for calculation.

### 4.11 Statistical Analysis

All results with n ≥ 3 were evaluated using one‐way Analysis of Variance (ANOVA), followed by Dunnett's multiple comparison test with GraphPad Prism 9 software. Significant differences were assumed at ^*^ *p* < 0.05; ^**^ *p* < 0.01; ^***^ *p* < 0.001; ^****^ *p* < 0.0001. Results with a smaller sample size (n < 3) were displayed as mean ± range.

## Conflicts of Interest

The authors declare no conflicts of interest.

## Supporting information




**Supporting File 1**: adhm71004‐sup‐0001‐SuppMat.docx.

## Data Availability

The datasets generated during the current study are available from the corresponding author on reasonable request.
