# Supplementary material for: Oxygen Supply of Islets of Langerhans by Photosynthetically Active Microalgae in Bioprinted Co‐Cultures Maintains Their Function in a Hypoxic Environment
Source: Adv Healthc Mater. 2026 Mar 8;15(19):e05927. doi: 10.1002/adhm.202505927 (PMC13206463; doi:10.1002/adhm.202505927)
Supplement: Supplementary file 1 — Supporting File 1: adhm71004‐sup‐0001‐SuppMat.docx. [file ADHM-15-0-s001.docx]

Supporting Information

Oxygen supply of Islets of Langerhans by photosynthetically active microalgae in bioprinted co-cultures maintains their function in a hypoxic environment

Finn Dani, Sarah Duin, Ashwini Rahul Akkineni, Susann Lehmann, Barbara Ludwig, Michael Kühl, Michael Gelinsky, Anja Lode^*^

^*^ Corresponding author: anja.lode@tu-dresden.de

**Table S1:** Components of standard INS-1 medium, given in vol%

| **Concentration** | **Component** |
| --- | --- |
| 85 % | RPMI 1640 |
| 10 % | HI-FBS |
| 1 % | 0.1 m Na-Pyruvate / 5 × 10^-3^ m 2-mercaptoethanol |
| 1 % | 0.2 m L-glutamine |
| 2 % | 1 m HEPES buffer |
| 1 % | 10 × 10^3^ U mL^-1^ penicillin / 10 mg mL^-1^ streptomycin |

**Table S2:** Components of standard medium for rat pancreatic islets, given in vol%

| **Concentration** | **Component** |
| --- | --- |
| 86.45 % | RPMI 1640 (no glucose) |
| 10 % | HI-FBS |
| 0.55 % | 1 m glucose |
| 2 % | 1 m HEPES buffer |
| 1 % | 10 × 10^3^ U mL^-1^ penicillin / 10 mg mL^-1^ streptomycin |

**Table S3:** Components of standard TP medium

| **Concentration** | **Component** |
| --- | --- |
| 96,8 % | Deionized H_2_O |
| 2 % | 1 m TRIS-base |
| 1 % | Salt solution (Table S4) |
| 0.1 % | Phosphate buffer (Table S4) |
| 0.1 % | Hutner’s trace element solution (Table S4) |

**Table S4:** Components of stock solutions for TP medium

| **Concentration** | **Component** |
| --- | --- |
| **Salt solution (in ddH_2_O)** | |
| 10 g/L | MgSO_4_ • 7 H_2_O |
| 5 g/L | CaCl_2_ • 2 H_2_O |
| 75 g/L | NaNO_3_ |
| **Phosphate buffer (in ddH_2_O)** | |
| 108 g/L | K_2_HPO_4_ |
| 56 g/L | KH_2_PO_4_ |
| **Hutner’s trace element solution (in ddH_2_O)** | |
| 50 g/L | Na_2_EDTA |
| 22 g/L | ZnSO_4_ • 7 H_2_O |
| 11.4 g/L | H_3_BO_3_ |
| 5.06 g/L | MnCl_2_ • 4 H_2_O |
| 4.99 g/L | FeSO_4_ • 7 H_2_O |
| 1.61 g/L | CoCl_2_ • 6 H_2_O |
| 1.57 g/L | CuSO_4_ • 5 H_2_O |
| 1.10 g/L | (NH_4_)_6_Mo_7_O_24_ • 4 H_2_O |
| ~16 g/L | KOH |


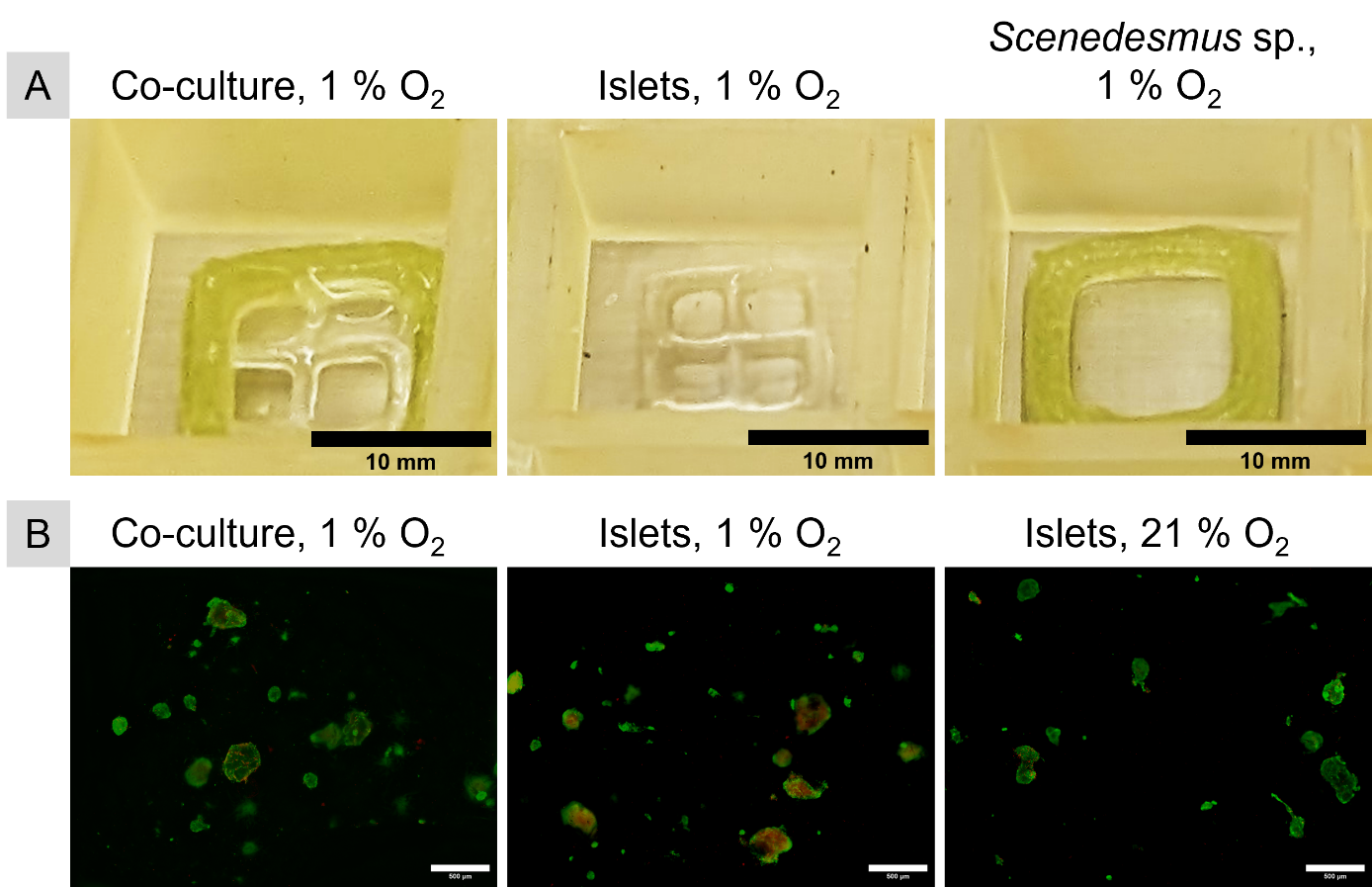


**Supplementary Figure S1:** **A)** Macroscopic images of rat pancreatic islet/Scenedesmus sp. co-cultures and respective mono-cultures on day 0 of the cultivation. **B)** Representative images of viability staining of rat islets in co-culture with Scenedesmus sp. in hypoxia (1% O_2_) and in mono-culture in hypoxia or normoxia (21% O_2_) (green: live cells, red: dead cells; scale bars: 500 µm).


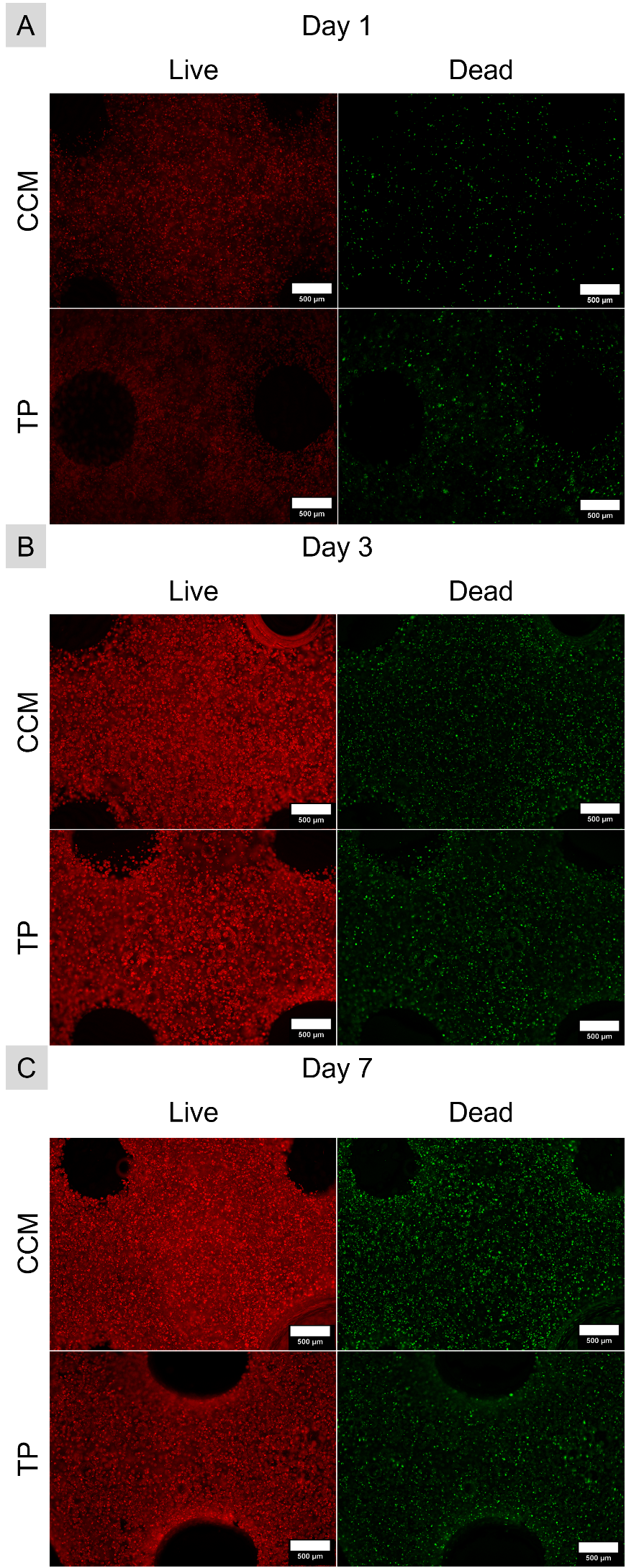


**Supplementary Figure S2:** Representative images of viability staining of Scenedesmus sp. in co-culture medium (CCM) or standard algal TP medium on day 1 **(A)**, day 3 **(B)** and day 7 **(C)** of cultivation under red-light illumination (green: live cells, red: dead cells; scale bars: 500 µm).


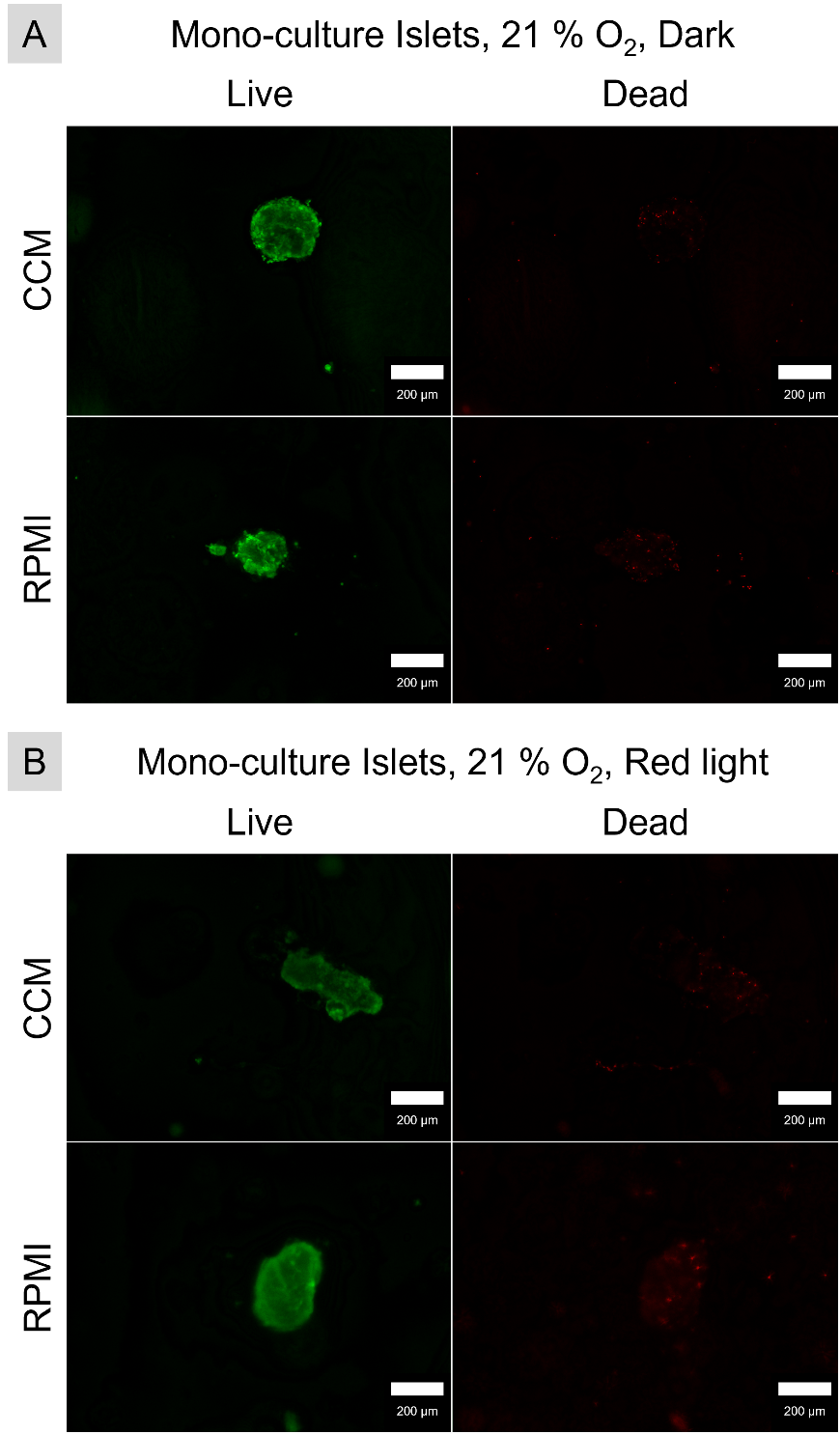


**Supplementary Figure S3:** Representative images of viability staining of rat pancreatic islets in the dark **(A)** and under red light illumination **(B)** after 4 days of cultivation in standard RPMI 1640-based culture medium (green: live cells, red: dead cells; scale bars: 200 µm).


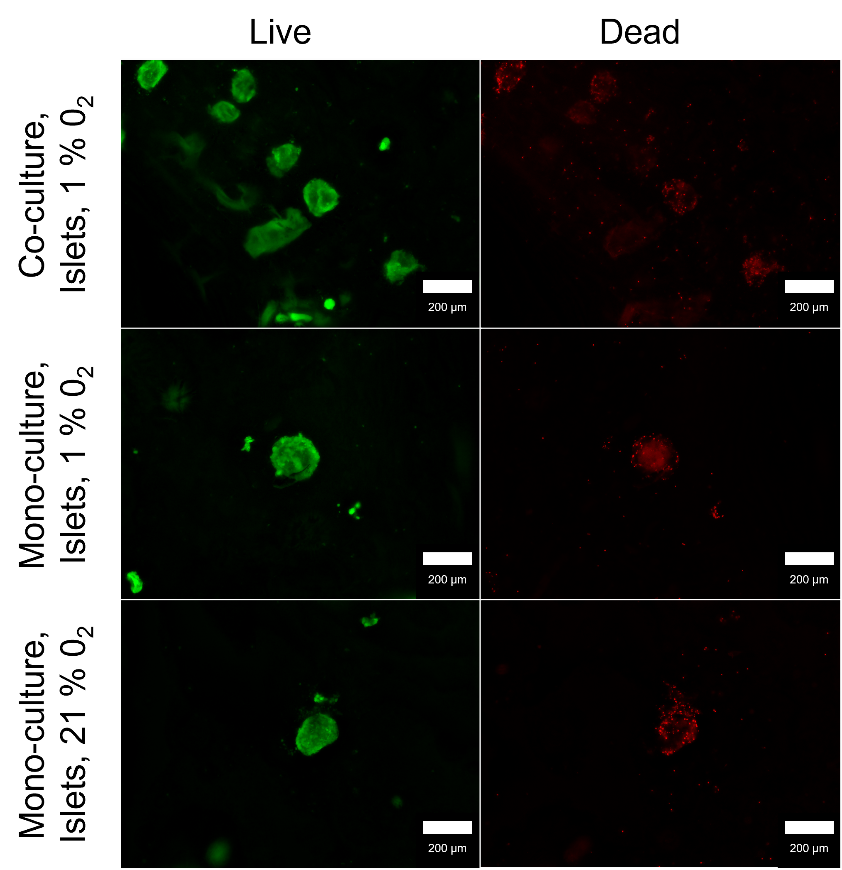


**Supplementary Figure S4:** Representative images of viability staining of rat pancreatic islets cultivated in co-culture with Scenedesmus sp. in hypoxia (1% O_2_) or mono-culture in hypoxia or normoxia (21% O_2_) after 4 days of cultivation in co-culture medium (CCM) (green: live cells, red: dead cells; scale bars: 200 µm).
